# The Quiet and Underappreciated Rise of Drug-Resistant Invasive Fungal Pathogens

**DOI:** 10.3390/jof6030138

**Published:** 2020-08-18

**Authors:** Amir Arastehfar, Cornelia Lass-Flörl, Rocio Garcia-Rubio, Farnaz Daneshnia, Macit Ilkit, Teun Boekhout, Toni Gabaldon, David S. Perlin

**Affiliations:** 1 Center for Discovery and Innovation, Hackensack Meridian Health, Nutley, NJ 07110, USA; Rocio.GarciaRubio@hmh-cdi.org; 2 Institute of Hygiene and Medical Microbiology, Medical University of Innsbruck, 6020 Innsbruck, Austria; cornelia.lass-floerl@i-med.ac.at; 3 Westerdijk Fungal Biodiversity Institute, 3584 CT Utrecht, The Netherlands; farnaz.daneshnia@gmail.com (F.D.); t.boekhout@wi.knaw.nl (T.B.); 4 Division of Mycology, University of Çukurova, 01330 Adana, Turkey; macitilkit@gmail.com; 5 Institute of Biodiversity and Ecosystem Dynamics (IBED), University of Amsterdam, 1012 WX Amsterdam, The Netherlands; 6 Life Sciences Programme, Barcelona, Supercomputing Center (BSC-CNS), Jordi Girona, 08034 Barcelona, Spain; tonigaes@gmail.com; 7 Mechanisms of Disease Programme, Institute for Research in Biomedicine (IRB), 08024 Barcelona, Spain; 8 Institució Catalana de Recerca i Estudis Avançats (ICREA), 08010 Barcelona, Spain

**Keywords:** *Candida glabrata*, *Candida parapsilosis*, *Candida tropicalis*, *Candida auris*, *Aspergillus terreus*, *Aspergillus fumigatus*, antifungal resistance mechanisms

## Abstract

Human fungal pathogens are attributable to a significant economic burden and mortality worldwide. Antifungal treatments, although limited in number, play a pivotal role in decreasing mortality and morbidities posed by invasive fungal infections (IFIs). However, the recent emergence of multidrug-resistant *Candida auris* and *Candida glabrata* and acquiring invasive infections due to azole-resistant *C. parapsilosis*, *C. tropicalis*, and *Aspergillus* spp. in azole-naïve patients pose a serious health threat considering the limited number of systemic antifungals available to treat IFIs. Although advancing for major fungal pathogens, the understanding of fungal attributes contributing to antifungal resistance is just emerging for several clinically important MDR fungal pathogens. Further complicating the matter are the distinct differences in antifungal resistance mechanisms among various fungal species in which one or more mechanisms may contribute to the resistance phenotype. In this review, we attempt to summarize the burden of antifungal resistance for selected non-*albicans* *Candida* and clinically important *Aspergillus* species together with their phylogenetic placement on the tree of life. Moreover, we highlight the different molecular mechanisms between antifungal tolerance and resistance, and comprehensively discuss the molecular mechanisms of antifungal resistance in a species level.

## 1. Introduction

Numerous fungal species, from yeasts and yeast-like fungi to molds, constitute human mycobiome and inhabit the gastrointestinal tract of healthy individuals [1]. However, the gut-resident fungi can translocate from the gut to the bloodstream and cause lethal invasive fungal infection (IFI) when the immune system is impaired [2]. Fungi profoundly affect human health. Based on global estimations, they cause 1.7 billion benign superficial infections, and IFI that is responsible for 1.5 million patient deaths annually [3]. The main causative species belong to the *Candida*, *Cryptococcus*, and *Aspergillus* genera. The incidence of fatal invasive fungal diseases is rising because of an increasing population at risk in developed countries, e.g., individuals with immunological deficiency, hematological malignancy, solid organ transplant recipients, and those with chronic obstructive pulmonary disease or exposed to continued corticosteroid therapy [4].

Although limited in number and chemical classes, antifungal treatments and/or prophylaxis are central to reducing comorbidities and mortalities caused by fungal infections. Yet it is considered as a driving force that replaces sensitive fungal species with other species exhibiting intrinsic and/or acquired resistance [5]. These emerging species are associated with longer hospitalizations, increased therapeutic failure, and increased costs, when compared to *C*. *albicans*, the most predominant fungal species causing bloodstream infection in humans [6,7]. Currently, clinical guidelines endorse treating IFIs caused by *Candida* and *Aspergillus* species by echinocandins and mold-active triazoles, respectively [8,9]. Underlying host conditions, antifungal pharmacokinetics and pharmacodynamics, and fungal attributes may alone or collectively contribute to therapeutic failure. Fungal factors resulting in antifungal resistance involve various subcellular mechanisms, including alteration of the drug target, overexpression of efflux pumps and drug target, and gross chromosomal changes [10].

Antifungals have different modes of action and belonged to three major classes, namely azoles (fluconazole, voriconazole, itraconazole, isavuconazole, and posaconazole, etc.), polyenes [amphotericin B (AMB)], and echinocandins (caspofungin, micafungin, and anidulafungin). Azoles disrupt fungal ergosterol production by binding to one of the critical enzymes (Erg11p) in the ergosterol biosynthesis pathway, which results in the accumulation of toxic sterols; polyenes bind to ergosterol and cause fungal cell death by forming pores on the cell membrane and disturbance of osmotic pressure; and echinocandins inhibit the biosynthesis of a key cell wall polymer, β-1,3-d-glucan, by blocking the catalytic subunit of glucan synthase enzyme, encoded by the *FKS* gene [10]. The modes of action and fungal cell fate depend on the cellular target, fungal species, and antifungal used. For instance, azoles are fungistatic against *Candida*, meaning that they do not kill the *Candida* cells but prevent cell division, while echinocandins exert fungicidal activity against *Candida* causing cell death. It is important to highlight the difference between tolerance and resistance. The former encompasses rapid cellular changes that lead to a transient (phenotypic) tolerance to the antifungal drugs, which is visible after 48 h, while the latter involves heritable genomic changes, ranging from point mutations to gross chromosomal changes resulting in permanent antifungal resistance, which is visible after 24 h (reviewed in [11]).

In the current review, we provide an overview of the epidemiology and molecular mechanisms of tolerance and resistance to antifungals of three most prevalent non-*albicans Candida* (NAC) species, namely *Candida glabrata*, *C*. *parapsilosis*, and *C*. *tropicalis*, the multidrug-resistant (MDR) *C*. *auris*; and most prevalent molds, namely *Aspergillus fumigatus* and *Aspergillus terreus*. Although, *Candida krusei* (*Pichia kudriavzevii*) shows intrinsic resistance to fluconazole, this species is not included in the scope of this paper, which is extensively described in a recent study published in 2020 [12]. Rather we focus on other NAC species ranked as the first to fourth most common cause of candidemia (except for *C*. *auris*). Additionally, biofilms exert intrinsic resistance against antifungals, but this topic has been extensively reviewed elsewhere [13,14,15,16,17] and will not be significantly addressed in the current paper.

## 2. Taxonomic Placement of Target Non-*albicans Candida* and *Aspergillus* Species

### 2.1. Candida

**Figure 1:**
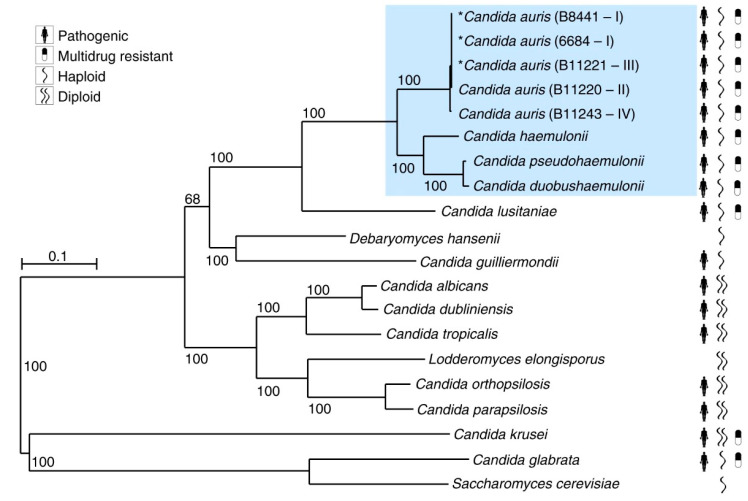
Phylogenetic tree of *Candida* species studied in the current review, i.e., *C. glabrata*, *C. parapsilosis*, *C. tropicalis*, and *C. auris* (highlighted in blue). This tree was constructed using maximum likelihood of 11,570 core genes based on 1000 replicates. Asterikes does not serve any specific defnitions. This figure was adopted permission from Munoz et al., 2018 [20].

Despite their shared name, *Candida* species do not constitute a genus in the phylogenetic sense. Indeed, when molecular data are used to place them in the Saccharomycotina phylogenetic tree, they are spread at different positions, intermingled with other non-*Candida* species [18]. The list of *Candida* species that causes candidiasis is long, with over 30 different species, although most of them are only rarely isolated from patients. Among the NAC pathogens covered in the current review, *C*. *parapsilosis* and *C*. *tropicalis* are relatively close to *C*. *albicans*, and belong to the *Lodderomyces* clade. Nevertheless, within this clade they belong to clearly different lineages that are separated by non-pathogenic species. Of note, *C*. *parapsilosis* belongs to a species complex that comprises other, less-prevalent pathogenic species that are hybrids such as *C*. *orthopsilosis* and *C*. *metapsilosis*, and for which hybridization has been proposed as a virulence emergence mechanism [19]. *Candida glabrata* is distantly related to *C*. *albicans*, being more closely related to the model yeast *Saccharomyces cerevisiae*, and belonging to a clade of yeasts that underwent whole-genome duplication (WGD) via hybridization approximately 100 million years ago [18]. In this post-WGD clade, only *C*. *glabrata* and some of its closest relatives (in the *Nakaseomyces* clade) can be considered regular opportunistic pathogens. Finally, *C*. *auris* is more distant from *C*. *albicans* than *C*. *glabrata* and belongs to the so-called *Metschnikowia* clade, which diverged earlier within the Saccharomycotina tree (Figure 1) [20]. The taxonomic classification and naming of Saccharomycotina yeasts is currently being revisited and will ultimately entered into the clinics. The fact that opportunistic *Candida* pathogens belong to such diverged clades indicates that their ability to infect human has emerged independently multiple times during evolution, which is further highlighted by the variable molecular mechanisms of virulence and differential antifungal susceptibility patterns [18,21,22].

### 2.2. Aspergillus

By contrast, the *Aspergillus* genus comprises more than 340 species [23], which are fungal saprophytes that are found in diverse ecological niches around the world. More predominantly, species within the *Fumigati* and *Terrei* sections are associated with clinical complications in humans, such as chronic and allergic pulmonary aspergillosis, saprophytic colonization, asthma with fungal sensitization and invasive aspergillosis (IA) [3].

The *Aspergillus* section *Fumigati* contains up to 63 species, although it is doubtful if some of them should be considered as species and possibly are synonymous with other species [24]. Species delimitation within this complex relies on several features that define five clades proposed based on them [25]: (I) *A*. *fumigatus*, (II) *A*. *lentulus* and *A*. *fumisynnematus*, (III) *A*. *fumigatiaffinis* and *A*. *novofumigatus*, (IV) *A*. *viridinutans*, *A*. *udagawae*, and other atypical strains; and (V) *A*. *hiratsukae*, *A*. *brevipes*, *A*. *duricaulis*, and *A*. *unilateralis*.

The *Aspergillus terreus* species complex is found in a wide variety of habitats, such as the soil, compost, and dust, but a specific niche is not known. The spectrum of diseases caused by these fungi covers allergic, chronic, invasive and disseminated forms of aspergillosis [26]. The section *Terrei* comprises 16 accepted species, namely *A*. *terreus* sensu stricto (*s*.*s*.), *A*. *alabamensis*, *A*. *allahabadii*, *A*. *ambiguus*, *A*. *aureoterreus*, *A*. *bicephalus*, *A*. *carneus*, *A*. *citrinoterreus*, *A*. *floccosus*, *A*. *iranicus*, *A*. *hortai*, *A. microcysticus*, *A*. *neoafricanus*, *A. neoindicus*, *A*. *niveus*, and *A*. *pseudoterreus* [27]. The production of aleurioconidia by *A*. *terreus s*.*s*., *A*. *carneus*, *A*. *flavipes*, and *A*. *niveus* seems to be a unique feature among the *Aspergillus* species. These morphologically distinct lateral conidia (aleurioconidia) are attached directly to hyphae and their function is as yet unknown [28].

## 3. Antifungal Tolerance Molecular Mechanisms and Its Implications as a Potential Therapeutic Option

Antifungal tolerance involves acute cellular responses to stressors, such as antifungals that threaten the integrity of the fungal cells. The fungal cells are constantly challenged by extrinsic stressors; hence, the cell wall and cell membrane are the two most important physical barriers responsible for cellular homeostasis. The viability and fate of fungal cell are largely determined by the abilities to sense the stress, integrate intracellular responses, and subsequently orchestrate a proper response. Stressors may lead to lethal consequences by destabilizing and degrading cellular proteins. However, molecular chaperones, such as HSP90, counteract the stressor effects by stabilizing critical and essential downstream client proteins, such as Mkcp and calcineurin, leading to stressor withstanding and tolerance [29,30]. Notably, master components involved in antifungal tolerance also play a role in virulence and biofilm formation [30]. These compensatory mechanisms orchestrate a rapid and appropriate response to stress, allowing the cell to “buy time” to acquire mutations in critical genes and/or undertake gross chromosomal changes, consequently leading to permanent resistance [31,32]. The acquisition of such mutations in genes associated with resistance may occur in the presence of specific mutations/absence of DNA repair mechanisms, such as mismatch repair (*MSH2*), resulting in increased antifungal tolerance and virulence [32,33]. Of note, the link between *MSH2*, in vitro tolerance, and clinical tolerance is uncertain [34,35].

**Figure 2:**
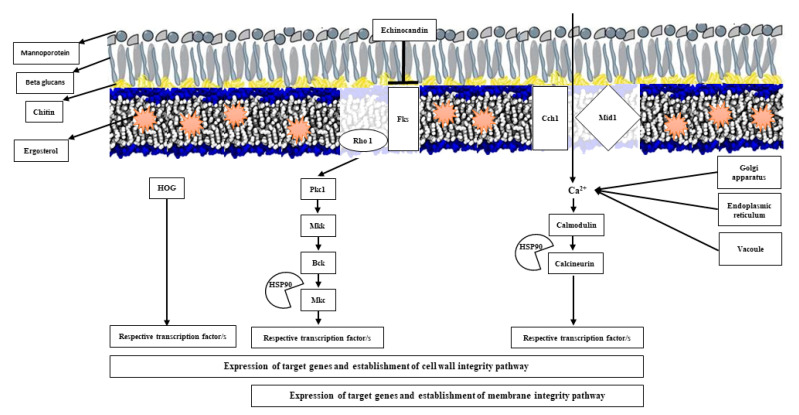
Mechanisms of antifungal tolerance. The mechanisms include rapid coordination of numerous signal transduction pathways that depend on the antifungal drug used. Echinocandin tolerance mechanism, known as the cell wall integrity pathway, involves protein kinase C (PKC), high-osmolarity glycerol (HOG), and calcineurin pathways, followed by the overexpression of chitin synthase, and *FKS1* and *FKS2* to compensate for the reduction of β-1,3-d-glucan level in the cell wall. Membrane integrity pathway orchestrates the azole tolerance pathways, which includes PKC and calcineurin pathways. As it is shown, HSP90 plays a critical role in antifungal tolerance by stabilizing the key regulatory proteins.

Echinocandins and azoles disrupt the cell wall and cell membrane integrity, respectively. Upon fungal cell exposure to echinocandins, cellular sensors detect the presence of drug molecules. This is followed by the engagement of main signal transduction pathways involved in stress adaptation and cell wall integrity, including protein kinase C (PKC), high-osmolarity glycerol (HOG), and calcineurin pathways, activation of appropriate transcription factors, and, finally, expression of response element genes, such as *FKS*, and *CHS2*, and *CHS8* (Figure 2) [36,37]. Activation of these pathways results in increased chitin levels in the cell wall, as a substitute for the reduced quantity or loss of β-1,3-d-glucan [37]. Interestingly, *A. fumigatus* displays paradoxical growth effect (PGE) when exposed to caspofungin, i.e., inhibition of growth at minimum inhibitory concentration (MIC) (0.5 µg/mL), but not above MIC (4 µg/mL). The inhibition of growth at MIC involves a relocalization of β-1,3-d-glucan synthase complex (Fks1p and Rho) from cell wall to vacuole, while continuous exposure to high MIC after 48 h results in returning of this complex to cell wall, which results in normal growth [38]. New lines of studies have found that a transcription factor, FhdA, plays an important role in PGE, which is involved in iron metabolism and mitochondrial respiratory function [39]. Importantly, addition of farnesol can block PGE in *A. fumigatus* when exposed to caspofungin [38]. Further, generally, azole tolerance results from an independent contribution of PKC and calcineurin pathways, leading to the activation of efflux pumps (Figure 1) [29,31]. Antifungal tolerance exerted by biofilm, however, does not prominently engage calcineurin and PKC pathways, in which the β-1,3-d-glucan matrix is significantly increased [40].

Tolerant cells exhibit the same level of minimum inhibitory concentration (MIC) as susceptible ones after 24 h incubation, therefore could be misidentified as susceptible isolates. If incubated for 48 h, however, tolerant cells can grow at a drug level higher than MIC, which is due to slow growth of tolerant cells in presence of antifungal drugs relative to drug-resistant isolates [11]. Of note, the level of tolerance is driven by the number of tolerant cells, which varies among isolates and could be measured quantitatively [11]. Some studies have shown the clinical implication of tolerant cells and observed that isolates with a high level of tolerant cells poorly respond to fluconazole when compared to isolates having medium- and low-tolerance cells [11,41]. Genes involved in tolerance, including the ones encoding HSP90 and calcineurin, are highly evolutionarily conserved such that a human ortholog of HSP90 is still functional in yeast [42]. Therefore, HSP90 and calcineurin inhibitors designed for immunosuppression in human are highly active against *C*. *albicans* and *A*. *fumigatus*. It is known that caspofungin and fluconazole are fungistatic in *A*. *fumigatus* and *C*. *albicans*, respectively [43]. However, in vivo and in vitro studies revealed that genetic impairment of HSP90 and/or calcineurin inhibitors potentiate the efficacy of fluconazole and caspofungin, rendering them fungicidal [43]. In addition, HSP90 inhibitors elicit aberrant biofilm morphology and restrict the dispersal and viability of yeast cells [40]. Even though acquisition of mutation in an antifungal drug target results in antifungal resistance independent of HSP90 and calcineurin, the effect of echinocandin against *C*. *glabrata* isolates harboring mutations in *FKS1* and/or *FKS2* can be potentiated when used in combination with HSP90 inhibitors [36]. Similarly, the combination of caspofungin and fluphenazine, a calmodulin inhibitor, potentiated the effect of caspofungin in *C*. *glabrata* isolates harboring prominent known *FKS* mutations and increased the survival of the infected *Galleria mellonella* relative to those treated with caspofungin alone [44]. These observations may suggest the fact that both tolerance mechanisms and *FKS* point mutations synergistically may contribute to echinocandin resistance in vitro and therapeutic failure in vivo.

Although HSP90 and calcineurin inhibitors may hold promise for future use in combination with antifungal drugs, mutations in *HSP90* and *CNA1*, the catalytic subunit of calcineurin, leading to resistance to the respective inhibitors, have been identified [45,46]. Further, human HSP90 inhibitors are pumped out of the fungal cell by efflux pumps [45] and their profound immunosuppressive effect is lethal in a murine model of invasive candidiasis [43] and places the host at high risk of secondary infection. This highlights the importance of developing of HSP90 inhibitors specific to fungi.

## 4. Antifungal Resistance Overview

Antifungal resistance is either acquired or innate (inherent). The former involves permanent resistance evolved during the course of antifungal therapy, while the latter is defined when a species intrinsically exhibits show elevated MIC values toward an antifungal. An example of innate resistance fungi is most *C*. *auris* isolates, which has been recently recognized as a globally emerging multidrug-resistant species [47]. An important consideration regarding the issue of susceptibility to antifungal drugs of different pathogens is that resistance can have different origins. On the one hand, different species have different intrinsic susceptibility to different antifungal drugs [21,22], which defines a shared trait of a species, but is more or less variable among strains. Here again, different species might have different potentials to adapt to different drugs, and hence, the acquired resistance is not entirely independent of the phylogenetic background. Below, we focus on the acquired antifungal resistance mechanisms, from single-nucleotide polymorphisms to gross chromosomal changes.

### 4.1. Point Mutations Leading to Antifungal Resistance

Molecular mechanisms of antifungal resistance vary depending on the class of antifungals. Azole resistance is primarily orchestrated by the upregulation of the gene encoding the drug target (*ERG11*) and those encoding efflux pumps belonging to the major facilitator superfamily (MFS), such as *MDR1*, and ATP-binding cassette (ABC) transporters, such as *CDR1* and *CDR2* [48]. MFS efflux pumps are composed of 12–14 transmembrane proteins transferring azoles out of the fungal cell using a proton motive force [48]. ABC transporters are composed of two transmembrane and two cytoplasmic nucleotide-binding domains, and use ATP to pump azoles and/or toxic metabolites out of the cell [49]. Upregulation of *ERG11*, *MDR1*, and ABC transporter genes occurs mainly as a result of gain-of-function (GOF) mutations in genes encoding zinc finger transcription factors (Zn_2_-Cys_6_), such as *UPC2*, *MRR1*, and *TAC1* (*PDR1* in *C*. *glabrata*). Although the transactivators are promising druggable targets, high-resolution structures are not available, except for Upc2p [50]. Modification of the azole drug target, Erg11p, is another prominent mechanism of azole resistance. Acquisition of mutations resulting in amino acid substitutions at specific positions near the heme-binding site, including Y132F, K143R, and G464S, lowers the affinity of Erg11p to azoles, with a subsequent azole resistance [51].

Echinocandins are not efflux pump targets and resistance to this class of antifungals mainly develops through acquisition of mutation in short specific regions of the *FKS1* and *FKS2* genes, within hotspot (HS) regions [52]. Echinocandin-resistant yeasts from the *Lodderomyces* and *Metschnikowia* clades carry accountable mutations in HS1 and HS2 of *FKS1*, while mutations in HS1 of *FKS1* and *FKS2* are the most prevalent causes of echinocandin resistance in *C*. *glabrata* [52]. Elevated MIC values vary depending on the position and the nature of the amino acid substitution [52,53]. Although sequencing of HS regions is the most convenient way of determining echinocandin resistance mechanisms, accountable mutations outside of the HS regions were recently identified, reinforcing the importance of sequencing the whole *FKS* gene [54].

The rare occurrence of AMB resistance lead to a limited number of studies dedicated to deciphering AMB resistance mechanisms. The limited studies available implicated a role of *ERG3*, *ERG2*, and *ERG6* as the possible mechanisms involved in AMB resistance [55,56,57]. Future studies are warranted to comprehensively examine AMB resistance mechanisms.

### 4.2. Gross Chromosomal Changes Leading to Antifungal Resistance

Beyond point mutations, gross genomic rearrangements have been reported to confer resistance to different antifungal drugs. Indeed, it has been suggested that such genomic rearrangements may commonly predate the appearance of point mutations [21]. This would explain why resistance rapidly appears in cell populations exposed to low densities of an antifungal. Some gross genomic re-arrangements, such as aneuploidy, occur at higher rates than a specific point mutation, particularly under stress conditions, and they seem to be well tolerated by the yeast cell [58,59]. Aneuploidy results in a combined down- or upregulation of several genes. This may allow survival in the presence of a drug and selection for the aneuploidy until a more favorable point mutation occurs. In this regard, azole resistance in *C*. *albicans* has been linked with a specific segmental aneuploidy leading to the duplication of *ERG11* and *TAC1* genes involved in ergosterol synthesis and drug efflux, respectively [59]. Genomic rearrangements have also been suggested to play a role in the adaptation of *C*. *glabrata* to stressful conditions, including exposure to antifungals [60]. However, similar chromosomal aneuploidies appear spontaneously in the *C*. *glabrata* cultivated under non-stressful conditions [61]. Further, recent whole-genome sequencing analysis failed to identify consistent links between aneuploidy involving genes associated with drug resistance and increased-resistance profiles [62,63]. *Candida parapsilosis* and *C*. *tropicalis* are even less studied in this regard. Hence, although it is established that aneuploidy plays a role in mediating drug resistance in *C*. *albicans*, the impact of this resistance mechanism in other species remains to be clarified. Finally, other gross genomic changes such as copy-number variation and loss of heterozygosity (LOH) (in heterozygous species such as *C*. *albicans* and hybrids of the *C*. *parapsilosis* clade), have also been proposed as possible mechanisms mediating rapid cell adaptation to antifungals [21,64].

### 4.3. Antifungal Resistance and Fitness Cost

Although resistance is a favorable trait for fungus in the presence of antifungals, considering the associated alteration of major cellular components, resistant isolates are typically less fit than their susceptible counterparts when examined in the absence of antifungal agent [45,65]. Small genomic changes, such as those leading to amino acid substitution, might be associated with a trivial fitness cost, while some gross chromosomal changes render resistant isolates more susceptible to killing by macrophage [45]. The exception is *C*. *glabrata* where GOF resistance mutations increase fitness by both protecting from drug through induction of the key drug efflux transporter but also by decreasing immune surveillance by macrophages (more details provided in Section 6.1) [66]. Although replacement of resistant isolates by susceptible ones in the absence of antifungal drugs is a rational assumption compatible with the evolutionary concept of natural selection [45], it is not always the case.

## 5. Fungal-, Host-, and Drug-Related Factors Facilitating the Emergence of Antifungal Resistance

The gastrointestinal (GI) tract is considered to be a major source of invasive candidiasis, as well as a barrier for the penetration of antifungal drugs, especially echinocandins [67]. Furthermore, fungal cells robustly produce biofilms inside the GI tract, further impeding the penetration of target cells at an infection site by antifungal drugs. On the other hand, the low permeability of echinocandins across the intestinal barrier necessitates the use of a high dose of echinocandins to attain a sufficiently high concentration in the GI tract so that the drugs would exert fungicidal activity [68]. At four times the humanized dosage of caspofungin (20 mg/kg), the fungal burden in the GI tract of mice dropped, which reduced dissemination to other organs. However, rebound was associated with the emergence of echinocandin-resistant strains harboring mutation in *FKS1*/*FKS2*, [69]. Some species, such as *C*. *glabrata*, which can cause intraabdominal candidiasis, show a pronounced immune evasion and reduced neutrophil influx, resulting in progression from peritonitis to abscesses [67]. Consequently, as discussed above, various factors associated with the host, pathogen, and antifungal drugs together facilitate the emergence of antifungal resistance [70]. Delivering drugs at an appropriate level at the site of infection is critical to achieve pharmacodynamic targets attainment to maximize clinical outcome. For intraabdominal infections, the recommended standard of care drugs, the echinocandin micafungin, fails to achieve sufficient levels during therapy, while newer related drugs in clinical development show superior penetration properties [71].

## 6. Epidemiology and Mechanisms of Antifungal Resistance in NAC and *Aspergillus* Species

### 6.1. Candida glabrata

*Candida glabrata* is a prominent cause of bloodstream infection (candidemia) worldwide and the second leading cause in some countries, including USA [72], Canada [73], Australia [74], and Scandinavian countries [75,76,77,78,79,80]. According to numerous epidemiological studies, the number of candidemia cases caused by *C*. *glabrata* exhibits a temporal increasing trend [80,81,82,83,84]. The elderly, patients undergoing abdominal surgeries, and those previously exposed to echinocandins and azoles are susceptible to acquiring candidemia caused by *C*. *glabrata* [85,86]. *Candida glabrata* is known for its significant tolerance of antifungal drugs [32] and it can rapidly develop resistance during the course of antifungal therapy, which ultimately leads to therapeutic failure [85,87,88,89,90,91,92,93,94,95]. Based on a recent worldwide study evaluating the burden of candidemia and antifungal resistance, the SENTRY Antifungal Surveillance Program, the incidence of fluconazole-resistant (FLZR) *C*. *glabrata* isolates has increased from 8.6% to 10.1% during 1997–2014, and Latin American and Asian Pacific countries noted the highest rate of fluconazole resistance (10.6% and 6.8%) [79]. Although echinocandin resistance is not common among the other *Candida* species (except for *C*. *auris*), this phenomenon is apparent in *C*. *glabrata*, with the worldwide prevalence ranging from 1.7−3.5% depending on the echinocandin drug tested [79]. More importantly, 5.5–7.6% of FLZR *C*. *glabrata* isolates reported by the SENTRY study are co-resistant to echinocandins and considered MDR [79]. At the institutional level, the prevalence of echinocandin resistance can vary significantly, reaching up to >13% at some centers [96].

**Figure 3:**
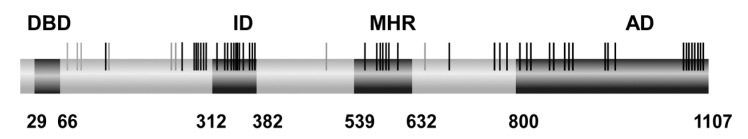
Pdr1p contains four domains. Numerous GOF mutations (black bars) can cause azole resistance. Adopted the permission from Ferrari et al., 2009 [98].

Except for a single study associating *ERG11^G315D^* with azole resistance [97], GOF mutations in *PDR1* appear to be a prominent factor driving azole resistance in *C*. *glabrata* in vitro and in vivo [98]. As already discussed, the expression of efflux pump genes, including *CDR1*, *CDR2* (*PDH1*), and *SNQ2*, is regulated by Pdr1p [99]. The protein is comprised of four domains, namely, DNA-binding domain (DBD), inhibitory domain (ID) (equivalent of a xenobiotic-binding domain in *Saccharomyces cerevisiae*), middle-homology domain (MHD), and activator domain (AD) (Figure 3) [100]. Once an azole reaches a cell, it binds to the MHD and activates *PDR1*, followed by interaction of *PDR1* and Gal11A on PDRE, RNA polymerase II recruitment, and the overexpression of downstream genes, such as efflux pumps [101]. GOF mutations in the regions encoding ID and AD disrupt the inhibition and induce the activation of *PDR1*, respectively, while those in a region encoding MHD obviate the need for xenobiotic activation (here, azoles) [98]. As discussed earlier, the GOF mutations in *PDR1* play role in virulence and immune evasion [102]. Further, overexpression of the transcription factor gene *CgSTB5* abrogates azole resistance by downregulating the expression of efflux pump genes (but not *SNQ2*) [103]. Interestingly, a small molecule, iKIX1, inhibits the interaction between *Cg*Gal11A and *Cg*Pdr1, and not only significantly reduces the fungal tissue burden in mice systemically infected with WT-*CgPDR1* and *CgPDR1^L280F^* isolates when used in combination with fluconazole, but also reduces the adhesion of *C*. *glabrata* in a mouse model of urinary tract infection when used alone [104]. In addition, according to a recent study, chemical and/or genetic inhibition of histone acetyltransferase, Cgn5p, is lethal in *C*. *glabrata* isolates harboring GOF mutations in *PDR1*; it showed a significantly reduced frequency of GOF mutations in CGN5-inhibited *C*. *glabrata* isolates compared with non-inhibited *C*. *glabrata* isolates in an evolutionary model of FLZR [100].

**Figure 4:**
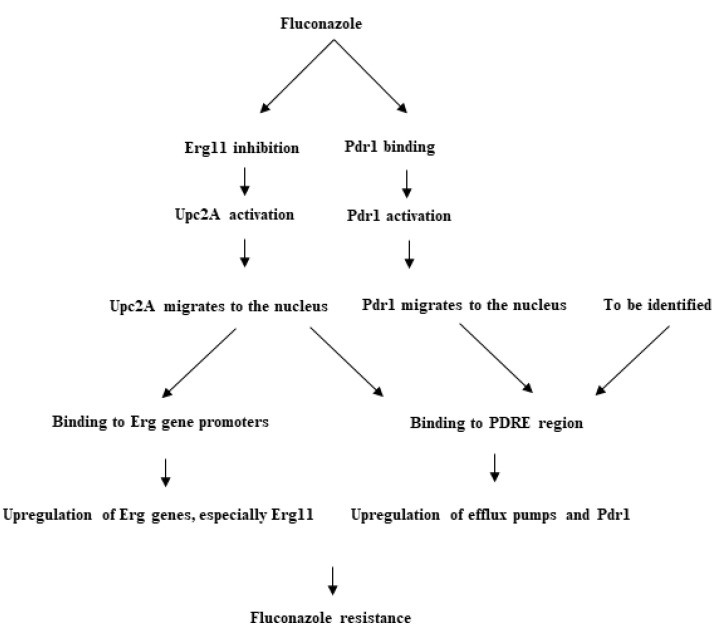
Fluconazole resistance is mediated by both Upc2p and Pdr1p in *C*. *glabrata*.

It was recently shown that the mechanism of azole resistance in *C*. *glabrata* involves a complicated circuitry of zinc cluster transcription factors other than Pdr1, such as Upc2A (Figure 3) [105,106]. Interestingly, double deletion of Upc2A (*upc2A*Δ) in both fluconazole-susceptible and FLZR *C*. *glabrata* isolates results in a 16-fold decrease of FLZ MIC, and downregulation of *CDR1*, *PDH1*, and *PDR1* upon induction by FLZ [106]. Indeed, Upc2A directly binds to the *CDR1* and *PDR1* promoters, leading to the overexpression of these genes and FLZR (Figure 4) [105]. However, *PDR1* and *CDR1* upregulation in the *upc2A*Δ isolate [105] may suggest that FLZR in *C*. *glabrata* is more complicated than currently thought, and may involve other zinc-cluster transcription factors.

Resistance to echinocandins appears to be more straightforward, and mainly associated with non-synonymous mutations in HS1 of *FKS1* and *FKS2*. S629P in Fks1, and S663P and F659deletion in Fks2p are the most prominent substitutions involved in both in vitro and in vivo resistance (Table 1). Of note, mutations occurring outside of these HS regions can also lead to echinocandin therapeutic failure [54]. Therefore, isolates displaying echinocandin resistance without known mutations in the HS regions may harbor non-synonymous mutations located anywhere in the *FKS* genes. Importantly, it has been documented that occasionally *C. glabrata* blood isolates carrying mutation in HS1-Fks1 (S629T) are fully susceptible to echinocandins, while the patient infected with such isolate showed therapeutic failure [107]. Therefore, combination of both antifungal susceptibility testing (AFST) and *FKS* sequencing can more precisely predict therapeutic failure when treating candidemia patients with echinocandins. Of note, this finding warrants further confirmation by larger studies and not all routine laboratories have direct access to both methods.

Recently, it was proposed that the presence of mutation(s) in a gene of DNA repair pathway, *MSH2*, increases the propensity of clinical *C*. *glabrata* isolates to acquire in vitro resistance to antifungal drug(s) [60]. This notion has been evaluated in French [136], Chinese [124], Spanish [35], and Indian [137] clinical isolates of *C*. *glabrata*, and it appears that the presence of a mutation in *MSH2* is associated with the genotype but not with the acquisition of antifungal resistance. Although, these clinical studies reported little or no resistance, which makes biological associations seems suspicious. Nevertheless, some *MSH2* mutations, but not all, are associated with antifungal multidrug resistance and future studies are warranted to identify the accountable mutations [32].

### 6.2. Candida tropicalis

*Candida tropicalis* is the primary cause of candidemia in India [138], Tunisia [139], and Algeria [140], the second cause of candidemia in Asian Pacific countries [141], and fourth cause worldwide [79]. It is considered to be the most virulent species after *C*. *albicans* [142] and like *C*. *glabrata*, this yeast can develop antifungal resistance during the course of antifungal therapy [125,126,143]. Candidemia patients infected with *C*. *tropicalis* show the poorest prognosis and highest mortality rate compared with patients infected with other NAC species [144]. Patients suffering from leukemia and neutropenia are considered to be highly susceptible to developing *C*. *tropicalis* candidemia [7]. The SENTRY study has noted a two-fold increase in the number of FLZR *C*. *tropicalis* isolates in the years 1997–2014, with the highest rate detected in Asian Pacific countries [79]. Indeed, significant increase of the incidence of FLZR *C*. *tropicalis* isolates has been noted by numerous institutional and nationwide studies, and ranges from 6.7% to 42.7% [95,145,146,147,148]. This could be associated with disproportionate azole use in the clinic. Surprisingly, according to studies conducted in Taiwan [146], Japan [147], Iran [149], and Turkey [150], almost 50% of candidemia patients infected with FLZR *C*. *tropicalis* isolates are azole naïve. Unfortunately, as is the case with *C*. *parapsilosis*, the vast majority of FLZR *C*. *tropicalis* isolates are identified in developing countries, hampering the efficacy of FLZ in these countries. Although, the global rate of echinocandin resistance remains low (0.5–0.7%), an increasing trend for echinocandin resistance has been noted for *C*. *tropicalis* in the years 2015–2016 [79].

As for other NAC species, MDR was reported in some studies and, strikingly, almost 1% of the Indian *C*. *tropicalis* blood isolates are resistant to the three major classes of antifungals [138]. Unlike *C*. *parapsilosis*, it appears that *ERG11* overexpression is a more prominent FLZR mechanism than efflux pump activity in *C*. *tropicalis* [151,152,153,154], and *MDR1* overexpression [152,155,156] is more prevalent than *CDR1* overexpression [152,156]. Surprisingly, FLZR *C*. *tropicalis* isolates lacking any accountable mutation in *ERG11* and not overexpressing efflux pumps were also identified, suggesting the involvement of other, unknown, mechanisms [156]. In one study, mutations in the promoter region of *UPC2* were identified but the authors never tested whether they could cause *UPC2* and *ERG11* overexpression and, in turn, azole resistance [157]. The presence of non-synonymous mutations in regulator genes, and their effect on azole resistance and target genes, were assessed by a limited number of studies. Considering the increasing number of FLZR *C*. *tropicalis* isolates, especially in developing countries, better understanding of the mechanism of azole resistance in this species is of paramount importance. Y132F is the most prevalent accountable amino acid change in Erg11p (Table 2) and is most frequently observed together with S154F; the latter does not confer azole resistance [152]. Various mutations linked to echinocandin resistance have been identified; S645P in HS1 of Fks1 is the most prominent amino acid change (Table 1). The mode of transmission of this species remains inconclusive, with some studies suggesting horizontal transfer from a contaminated hospital environment [158,159,160], while others assuming colonization of individuals outside of the clinic, via agricultural and crop sources (see Section 7), followed by subsequent dissemination in the hospital [146].

### 6.3. Candida parapsilosis

*Candida parapsilosis* is the second leading cause of candidemia in Latin American countries [174], and in some Asian [83,175,176,177,178], European [179,180,181], and African countries [140,182], and the third cause of candidemia worldwide [79]. Neonates, patients receiving total parenteral nutrition, and those with central venous catheters (CVC) are most prone to developing candidemia caused by *C*. *parapsilosis* [183]. Recently, the SENTRY study revealed that Latin American (4.6%) and Asian Pacific countries (0.6%) have the highest and lowest percentage of FLZR *C*. *parapsilosis* isolates, respectively [79]. However, this is beyond the scale reported from a South African multicenter nationwide study, according to which more than 50% of *C*. *parapsilosis* isolates are FLZR, with 44% among these cross-resistant to VRZ (VRZR) [182]. Furthermore, a Korean single-center study reported a significant increase in the prevalence of FLZR among 2015–2016 isolates when compared to 2011–2015 isolates (14.3% vs. 0.9%, respectively) [184]. Unfortunately, this wave of FLZR *C*. *parapsilosis* isolates has also been observed in Kuwait [166], USA [165,185], Brazil [167,168], South Korea [164], India [169], South Africa [182] and Turkey [170]. The high rate of FLZR for a species that used to be susceptible to azoles may have arisen from the disproportionate use of azoles in the hospital [170,182]. The notable increase in the prevalence of FLZR *C*. *parapsilosis* isolates could be a major threat in developing countries, in which the vast majority of candidemia cases are treated with FLZ [138,169,182,186]. By contrast, according to the SENTRY study, echinocandin resistance is a rare phenomenon among *C*. *parapsilosis* isolates (up to 0.1%) [79].

Evaluation of FLZR *C*. *parapsilosis* isolates revealed that FLZR mechanisms involve *ERG11* mutations, and upregulation of *CDR1* and *MDR1*, and in few cases *ERG11*, which might arise from GOF mutations in the respective zinc cluster regulators, *TAC1*, *MRR1*, and *UPC2*, accordingly (Table 2). Although some studies link the substitutions R478K, G583R, L779F, and K873N in Mrr1p to azole resistance [165,187], the role of other mutations in the aforementioned regulators is largely unknown. Surprisingly, the overexpression of *CDR1*, *MDR1*, and *ERG11* in the absence of any mutations in the corresponding regulators [165,185], and 2-fold dilution decrease in FLZ MIC values when either *CDR1* or *MDR1* are deleted [185], are indications for the involvement of other mechanisms. Therefore, a comprehensive transcriptomic and proteomic analysis of FLZR *C*. *parapsilosis* isolates may allow a better understanding of the possible mechanisms of azole resistance in *C*. *parapsilosis*. Y132F and K143R are presumably the most frequently identified amino acid changes causing FLZR and/or VRZR, and we noticed that isolates harboring Y132F in Erg11p are significantly associated with a mortality rate that is higher than that associated with Y132F+K143R isolates [170]. Beyond the common paradigm of *ERG11* and efflux pump involvement in azole resistance, whole genome sequence analysis showed that a laboratory-driven, posaconazole-resistant *C. parapsilosis* isolate harbored an amino acid substitution in Erg3, R135I, which is believed to confer azole resistance and prevent the formation of toxic sterol intermediates [188].

A naturally occurring polymorphism in HS1 of *FKS1* (P660A) in *C*. *parapsilosis* results in elevated echinocandin MIC values in this species [189], yet an effect on the drug–target interaction seems weak and the infected candidemia patients treated with echinocandins show a favorable clinical outcome compared to those treated with FLZ [190,191]. Interestingly, despite the identification of clinical echinocandin-resistant *C*. *parapsilosis* isolates, no other mutations in HS1 and HS2 of *FKS1* were identified to date. Most recently, we evaluated a large collection of Turkish *C*. *parapsilosis* blood isolates (2007–2019) [129]. For the first time, we identified four isolates that were resistant to micafungin and carried R658G in HS1 of FKS1 (Table 1). Interestingly, three of those isolates represented the same genotype, were also further resistant to FLZ, and carried Y132F+K143R in Erg11p [129]. This represented an unprecedented clonal expansion of MDR *C*. *parapsilosis* isolates [129]. *Candida parapsilosis* is well-known for biofilm production on biotic and abiotic surfaces, and the hands of the healthcare workers are considered as one of the major sources of bloodstream infection [183]. Consequently, antifungal-naïve patients might acquire antifungal-resistant *C*. *parapsilosis* isolates from the hospital environment, which may in turn result in therapeutic failure. Therefore, strict adherence to hygiene and CVC removal could greatly reduce *C*. *parapsilosis*-associated candidemia.

### 6.4. Candida auris

**Figure 5:**
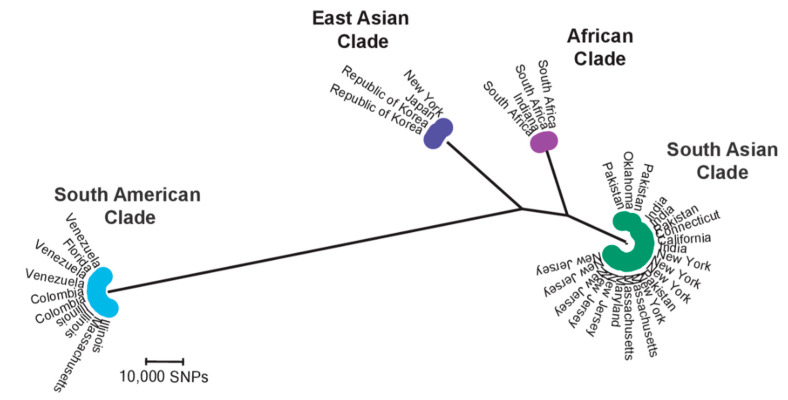
Whole-genome sequence analysis of the US *C*. *auris* (up to 2018) isolates reveals the existence of four major clades. Isolates representing all identified clades have been recovered from the U.S. The Iranian clade is not shown in this figure. Adopted the permission from Chow et al., 2018 [201].

*Candida auris* was described for the first time in 2009, causing an ear infection in a Japanese patient [192]. However, it soon became one of the most worrisome MDR pathogenic fungal species known, causing infection in over 35 countries on six continents [193]. This species is the third to fifth common cause of candidemia in South Africa [194] and India [138], respectively. Of note, in some hospitals in India, *C*. *auris* is the second most common cause of candidemia [195]. One of the most dramatic examples of the dominance of this species was documented in a course of a nationwide South African candidemia study, with *C*. *auris* identified as a rare cause of candidemia in 2009 but becoming the third most common cause of candidemia 7 years later [194] and on its way to become the leading candidemia cause. Its ability to persistently colonize the skin, hospital equipment, and environment [196], and survival on plastic surfaces for 4 weeks [197], combined with the inefficiency of the currently used disinfectants [198] necessitates intense infection control measures, such as those recommended for challenging bacterial species that cause nosocomial outbreaks, such as methicillin-resistant *Staphylococcus aureus* and *Clostridium difficile* [196]. Several hypotheses have tried to elucidate the simultaneous worldwide emergence of *C*. *auris* [199,200], yet its recent bizarre dominance remains enigmatic. Whole-genome sequencing revealed that the identified clinical and environmental isolates belong to four clades (clusters) representing geographical origin of the isolates, namely, the South Asian clade (clade I), East Asian Clade (clade II), South African Clade (clade III), and South American clade (clade IV) (Figure 5) [172]. Of note, according to an updated whole-genome study, a single isolate of Iranian *C*. *auris* potentially represents a fifth clade (not shown in Figure 4), consolidated by hundreds of thousands of base–pair differences with the closest clade (II) and the notion that the infected individual did not travel outside of Iran [193]. The concept that isolates from those clades have been detected in the healthcare setting hundreds to thousands of kilometers apart [130,172,201] suggests clonal expansion of *C*. *auris*, possibly via travel [193]. Catheter insertion, recent surgery, and previous exposure to antifungals are among the potential risk factors for the development of infection caused by *C*. *auris* [172,194].

Antifungal susceptibility profiles vary depending on the clade [131,172,173,195], with the drug resistance reaching up to 7%, 35%, and >90% against echinocandins [172], AMB [172], and fluconazole [131], respectively. Similar to *C*. *glabrata*, resistance against two or three major classes of antifungals is frequently observed [131,172]. Amino acid changes in Erg11p are closely associated with resistance and seems to be clade-specific, where clade III is prone to harbor F126T; Y132F is most prevalent in clade IV [172]; and Y132F and K143R are prominent in clade I (Table 2) [131]. Of note, according to a recent study examining the South Korean isolates, only a small proportion of FLZR isolates (3/38, 7.8%) harbor K143R and the remaining isolates lack accountable mutations [173]. This underscores the involvement of other, yet to be identified pathways, such as efflux pumps, with the emphasis on *CDR1*. *CDR1* plays a prominent role in azole resistance, as *CDR1* disruption significantly decreases the MIC value to azoles containing target site amino acid substitutions [202]. Furthermore, *CDR1* expression in *C*. *auris* 5–25 min post-exposure to fluconazole is 14.4–6.7 times higher than that in *C*. *glabrata* [203]. Since HSP90 inhibitors do not abrogate the azole resistance conferred by *CDR1* overexpression [204], it is not unreasonable to assume an involvement of *TAC1* overexpression, achieved via GOF mutations, in the resistance. Therefore, profiling and evaluation of GOF mutations in a transcriptional regulator of efflux pumps may further elucidate the azole resistance mechanisms. Moreover, *CDR1* plays an important role in the intermediate and late stages of biofilm growth, and inhibiting *CDR1* causes a 4–16-fold decrease in the FLZ MIC values [205]. Indeed, consistent with this hypothesis, the most recent study found that GOF mutations in *TAC1* are other players implicated in azole resistance in *C. auris* [206].

Although constitutive overexpression of *ERG11* has been observed in some isolates [131], this phenomenon is an unlikely player in azole resistance. Further, sectional genomic duplication (12–153 kb), with the largest occurring in clade III, *ERG11* is associated with azole resistance and/or elevated MIC values [20,207]. Echinocandin resistance is mainly associated with a substitution of serine 639 to proline (S639P), tyrosine, or phenylalanine (HS1 of *FKS1*). Among these, S639P is the most prominent amino acid change (Table 1) [132].

Finally, the AMB resistance can be a combination of overexpression [20] and/or non-synonymous mutations in a number of genes, and more studies are warranted to identify the role of these mutations in resistance [20,172].

### 6.5. Aspergillus fumigatus

*Aspergillus fumigatus* is the principal causative agent of human aspergillosis, accounting for more than half of all isolates in most studies [208,209]. Azole drugs are the main antifungal compounds used both in agriculture and the clinical setting, and the emergence of azole resistance is rising and spreading worldwide [210,211]. Based on a prospective multicenter international study involving 19 countries, the prevalence of azole-resistant *A*. *fumigatus* is 3.2% [212].

Generally, azole-resistant isolates are acquired via two routes. In the clinical setting, azole resistance may develop during long periods of azole treatment. It is associated with single-point mutations in a lanosterol-14-α steroldemethylase gene (*Cyp51A*), encoding a key protein in the ergosterol biosynthesis pathway, that lead to amino acid changes (G54, G138, P216, M220, and G448) [213]. Alternatively, extended use of demethylation inhibitors (DMIs) in agriculture is associated with tandem repeat (TR) integrations of different sizes in the *Cyp51A* promoter, followed, or not, by point mutations in the gene (TR34/L98H, TR46/Y121F/T289A, and TR53) [4]. Hence, azole selective pressure elicits the development of different azole resistance mechanisms and also different azole susceptibility patterns. Apart from azole drug target, some studies have indicated the emergence of notable number of azole-resistant *A. fumigatus* isolates displaying WT CYP51A [214,215] for which the azole resistance phenotype was corroborated by the overexpression of efflux pumps, especially Cdr1B [214]. Moreover, acquisition of various mutations in Hmg1 [216] HapE [217] also confer azole resistance in *A. fumigatus*.Other species of the *Aspergillus* section *Fumigati* that are also human pathogens show azole resistance, such as *A*. *lentulus*, *A*. *viridinutans*, *A*. *fumigatiaffinis*, and *A*. *fischeri* [218,219]. Although rarely used as a main treatment among patients suffering invasive aspergillosis, recent studies have revealed that mutation in HS1-FKS1 [135] and also changes posed to the microenvironment of β-1,3-d-glucan synthase in *A. fumigatus* can result in echinocandin resistance [220].

### 6.6. Aspergillus terreus

In the past, infections caused by *A*. *terreus* species complex were classified as rare, but their clinical incidence has recently increased. According to a prospective international multicenter surveillance study, the prevalence of *A*. *terreus* species complex among patients with mold-positive cultures is 5.2%, attributed *A. terreus s.s.* (86.8%), followed by *A. citrinoterreus* (8.4%), *A. hortai* (2.6%), *A. alabamensis* (1.6%), *A. neoafricanus* (0.2%), and *A. floccosus* (0.2%) (221). Frequent occurrences are noted at certain geographic locations, such as Innsbruck (Austria) and Houston (TX, USA). Of special concern is the high mortality of disseminated disease caused by this species [221]. The at-risk population for infections caused by *Aspergillus* section *Terrei* is the same as that for individuals suffering from *A. fumigatus* diseases, and comprises mainly immunocompromised individuals. However, non-immunocompromised individuals may also be affected [221].

*Aspergillus terreus* species complex holds an exceptional position within the aspergilli, as it displays polyene resistance in vitro and in vivo [222]. Generally, AMB MIC values range from 0.125 to 32 mg/L. The underlying AMB resistance mechanisms of *A. terreus* are only partly understood and are multifaceted [222,223]. AMB resistance seems to be related to basal superoxide dismutase activity and an enhanced oxidative stress response in *A*. *terreus*.

According to a recent study, approximately 5% of *A*. *terreus* *s*.*s*. isolates are resistant to posaconazole in vitro [224]. The prevalence of resistance differs geographically, and ranges from 0% in the Czechia, Greece, and Turkey, to 13.7% in Germany. The highest rates of resistance are observed in Austria, Germany, and the UK. Azole resistance in *A. terreus s.s.* is associated with mutations in the *Cyp51A* gene. In the Cyp51A protein, M217 position correlates with the posaconazole-resistant phenotype, with substitutions M217T and M217V reported [224] (Table 3). By contrast, azole resistance among cryptic species is rare, and observed only in *A. citrinoterreus* and *A. alabamensis.*

## 7. Role of Agriculture in the Development of Resistance

Azole drugs are the only class of compounds that are used both in agriculture and in the clinical setting [211]. In the context of agriculture, they are extensively used for crop protection, preservation of the yield and quality of crops against plant fungal diseases, and prevention of contamination by yeasts (*Candida* spp., *Trichosporon penicillatum*, and *Cryptococcus* spp.) and filamentous fungi (*Aspergillus* spp., *Fusarium* spp., and *Alternaria* spp.) during the pre- and post-harvest periods. The global pesticide use increased significantly during the years 2012–2016, with the highest use in Asia, 2 M tons collectively (52%); followed by America, with 1 M tons (32.7%); Europe, with 477 K tons (11.6%); Africa, with 95 K tons (2.3%); and Oceania, with approximately 55 K tons (1.4%) [237]. The increased use of antifungals in agriculture in recent years has paralleled the detection rate of fungicide-resistant pathogenic fungi. As *A. fumigatus* spores are ubiquitous in the environment, and environmentally acquired azole-resistant isolates, especially those in compost heaps [238], may exhibit in vivo and/or in vitro resistance to medically important azole drugs, such agricultural pesticides may threaten human health by exposure through contact, inhalation, or ingestion of contaminated food or water.

The hypothesis that an environmental source of resistant *A*. *fumigatus* isolates could underpin the emergence of azole resistance is supported by the identification of primary IA cases caused by azole-resistant *A*. *fumigatus* in patients who have never been treated with azoles [239]. Since environmental isolates harboring TR resistance mechanisms are identified in azole-naïve patients on five continents, and the same resistance mechanism was identified in environmental isolates treated with MDIs that share a higher genetic similarity with wild-type isolates, this strongly suggests that clinically acquired azole-resistant isolates are primarily acquired from the environment [4]. A recent study, however, through the use of whole-genome sequencing identified that azole-resistant *A. fumigatus* isolates harboring TR can also develop during the course of antifungal treatment, which shows the same genotype as the initial azole-susceptible *A. fumigatus* isolates [240]. It seems that the emergence of azole-resistant fungus in the clinic with the environmental source is not exclusive to molds. Recently, the same phenomenon was observed for *C*. *tropicalis* and 55.2% of azole-resistant isolates were recovered from patients never treated with azoles [146]. Interestingly, multi-locus sequence typing (MLST) revealed that the clinical azole-resistant isolates share a high degree of similarity with an azole-resistant isolate recovered from fruit, indicating an increasing danger of acquisition of environmental azole-resistant fungi that represent a wide spectrum of species, ranging from molds to yeasts [146].

## 8. Genomic Tools for Early Diagnosis of Resistance

AFST is a popular culture-based method of analysis. It is a phenotypic approach for the visual determination of the susceptibility of a fungal species to a specific antifungal, by reporting the MIC value. MIC is the lowest concentration of an antifungal that results in growth inhibition of a fungal species when compared to a positive control. AFST broth microdilution protocols are standardized by the Clinical Laboratory Standard Institute (CLSI; (http://www.clsi.org) and European Committee for Antimicrobial Susceptibility Testing (EUCAST; http://www.eucast.org/). Although AFST plays a central role in patient management by aiding the prescription of an appropriate antifungal, it is time-consuming and data interpretation varies depending on the protocol used and between laboratories.

In some cases, sequencing of drug target genes is a better predictor of patient outcome than AFST [86]. Consequently, various polymerase chain reaction (PCR)-based techniques have been developed for the identification of mutations in drug target genes, facilitating timely administration of an appropriate antifungal [241]. Any PCR-based or hybridization-based method that is able to detect the presence of single-point-mutations or genetic variations can be used to test for the existence of a set of known resistance-conferring mutations [242]. Such approaches are highly sensitive, and require a low amount of input DNA that does not need to be of the highest quality. However, these methods are designed to target a set of known resistance-conferring mutations. Hence, a negative result does not always imply the absence of resistance, they are best used to confirm known prominent mutations conferring antifungal resistance. Whole-genome sequencing or targeted sequencing of genomic regions known to confer resistance can be used for the detection of genomic alterations that potentially confer resistance in an isolate of interest. Although the obtained data should be crosschecked against a catalogue of known resistance-conferring mutations, these approaches can potentially uncover new variations (e.g., by assessing the potential impact on proteins encoded by the mutated genes), and they do not need to be re-designed every time the catalogue is expanded [242]. Nevertheless, although these approaches are promising, they have different limitations that delay their introduction in the clinic. The associated costs, required expertise, need for a high amount of template DNA, difficulty of direct probing clinical samples, and requirement for on-site technology are some of the challenges that need to be addressed [242,243].

## 9. Future Perspectives

The increasing number of fungal species resistant to antifungals and the emergence of MDR fungal species parallels with the global increase in the use of DMIs pose a serious threat to patient outcome, especially those in developing countries where azoles are the main antifungal used to treat invasive fungal infections. Therefore, epidemiological studies and constant monitoring of the burden of antifungal resistance on worldwide scale should be coupled to revisiting the antifungal stewardship protocols in both environment and clinics. Moreover, broadening our understanding about the mechanisms of antifungal resistance not only allows designing more rapid molecular techniques to rapidly diagnose antifungal resistance but also may lead to designing more efficient new fungal-specific antifungal drugs given the high genetic similarity of fungi with human. Therefore, comprehensive and species-specific multiomics studies and high-resolution structural approaches play an integral role in this context. Introduction of antifungal drugs showing optimal clinical profiles into the clinic and extending research to identify naturally occurring secondary metabolites showing promising antifungal drugs are more than ever needed. New online platforms, such as www.theyeasts.org, encourages researchers throughout the world to deposit the microbiological and therapeutic failure data of mutations occurring in drug target and efflux pump regulators allowing clinicians to promptly predict the possible MIC values of any mutation and if they can cause therapeutic failure. We hope that the rise of technical advances will accompany the extensive international collaboration that is needed to tackle the pressing challenge of antifungal resistance.

## Figures and Tables

**Table 1:** Mutations involved in echinocandin resistance and their in vitro and in vivo impact.

**Species**	**FKS1-HS1**	**MIC (µg/mL)**			**FKS2-HS1**	**MIC (µg/mL)**			**Therapeutic Failure**			**Reference**
	**Mutation**	**CSP**	**MCF**	**AND**	**Mutation**	**CSP**	**MCF**	**AND**	**CSP**	**MCF**	**AND**	
*C. glabrata*	*fks1*Δ + E655K	8	16	8	Y657del + F659Y	0.25–0.5	0.25	1		*fks1*Δ + E655K		[85,86,87,88,89,90,92,93,94,95,107,108,109,110,111,112,113,114,115,116,117,118,119,120,121,122,123,124]
	F625S	2	0.25	1	F659L	1–2	0.03–0.06	0.06–0.12				
	F625C	0.12	NA	NA	F659del	0.06–>32	0.12–4	0.12–4		F659del		
	F625I + P667T	4	2	0.25	F659Y	0.5–2	0.0.12–25	0.25–1		F659Y		
	S629P + S663F	1–32	4	0.5–2	F659S	0.25–1	0.03–0.25	0.06–1		F659S		
	I627V	0.06	0.25	0.03	F659V	2	0.25	0.5	F659V			
	S629P + D666V	0.12–4	0.5–1	0.12–2	F659S + L664V	4	0.5	1				
	S629P	0.06–>16	0.06–>16	0.5–8	F659Del + D666N	16	2	>4	S629P	S629P	S629P	
	S629T	-	0.016	0.064								
	R631G	0.12–0.5	0.25–0.5	0.06–0.5	F659S	1	0.25	1				
	R631G + D666V	0.25	0.5	0.5	F659S + S663A + D666E	4	0.25	1				
					S663P	0.06–>32	0.125–>16	0.25–>8	S663P	S663P	S663P	
	D632H	2	0.5	0.5	S663F	0.25–4	0.125–4	0.5–4	S663F			
	D632Y	0.12–2	0.06–0.5	0.25–0.5	L664R	1	0.06–0.12	0.12				
	D632V	0.12–2	0.25–2	1–2	R665G	0.12–0.5	0.25–1	0.06–1	D632V			
					D666H	0.5	0.06	0.12				
					D666E	1	0.06	0.25				
					D666N	0.5–2	0.06	0.12–0.25				
					P667H	0.25–2	0.12–0.25	0.25–2				
					P667T	2	0.015	0.25				
	**FKS1-HS1**	**MIC (µg/mL)**			**FKS1-HS2**	**MIC (µg/mL)**			**Therapeutic Failure**			
	**Mutation (Frequency)**	**CSP**	**MCF**	**AND**	**Mutation (Frequency)**	**CSP**	**MCF**	**AND**	**CSP**	**MCF**	**AND**	
*C. tropicalis*	F641L	1	0.5	0.5	NF	NA/WT	NA/WT	NA/WT	F641L			[90,91,95,125,126,127,128]
	F641S	4–2	1	1	NF	NA/WT	NA/WT	NA/WT	F641S			
	L644W	2	NA	NA	NF	NA/WT	NA/WT	NA/WT				
	S645P	4–>32	0.5–2	0.25–4	NF	NA/WT	NA/WT	NA/WT	S645P	S645P		
	R647G	NA	0.25	0.06	NF	NA/WT	NA/WT	NA/WT	R647G			
*C. parapsilosis*	R658G		>8	2–4	NF	NA/WT	NA/WT	NA/WT	R658G			[129]
*C. auris*	S639F	4–16	16	8	NF	NA/WT	NA/WT	NA/WT	S639F			[130,131,132,133,134]
	S639Y	8	8	8	NF	NA/WT	NA/WT	NA/WT				
	S639P	>16	8	8	NF	NA/WT	NA/WT	NA/WT				
*A. fumigatus*	F675S	2	2	ND	NF	NA/WT	NA/WT	NA/WT		F675S		[135]

**Table 2:** Mutations involved in azole resistance and their in vitro and in vivo impacts.

Species	Protein	Mutation	MIC (µg/mL)				Therapeutic Failure		References
			FLZ	VRZ	ITZ	PSZ	FLZ	VRZ	
*C. tropicalis*	Erg11p	P56S	8–≥64	0.125–16	≥16	ND			[140,141,145,146,147,148,149,150,151,152,153,154,155,156,157,161,162,163]
		P56S + V234F	8	1	0.5	ND			
		Y132F	8–>256	0.5–>32	0.25		Y132F	Y132F	
		Y132F + S154F	2–≥256	0.125–8	0.25–2	0.12–1	Y132F + S154F		
		Y132F + F145L + S154F	≥256	≥8	NA	1			
		V125F	8						
		Y257H	8–32						
		V234F	8	1	0.5				
		G464S	2–64	0.12–2	0.12–2	0.25–1	G464S		
		K143R	64–>64	4–8	1->8	1–4			
		L333I	4	0.5	1	0.25			
		G464D	>64	>8	ND	ND	G464D	G464D	
		Δ44aa + D275V + P511A	>64	>8	ND	ND			
	Upc2p	Δ301–304 (AQSP) + Q320PPQ (Proline insertion)	16	1	2	0.5	Δ301–304 (AQSP) + Q320PPQ		
		T241A	8	0.06	0.25	ND			
		Q340H+T381S	64	4	16	ND			
	Mrr1p	T255P	8	1	0.5	ND			
		A647S	8	1	0.5	ND			
	Tac1p	R47Q + N164I	8	1	0.5	ND			
*C. parapsilosis*	Erg11p	Y132F	2–>256	≤0.03–2	≤0.03–0.25	<0.015–0.125	Y132F		[151,164,165,166,167,168,169,170,171]
		K143R	4	≤0.03–0.5	≤0.03–1	<0.015–0.25			
		Y132F + K143R	32–>32	0.06–4	ND	ND	Y132F + K143R		
		G458S	16–>32	0.5–1	ND	ND			
		G307A + Y132F	16–>32	0.5–2	ND	ND			
		Q250K + G458S	16	0.25	ND	ND			
		G458S + T519A	16	0.5	ND	ND			
	Mrr1p	G_53A	4–16	NI	ND	ND			
		-102_101-insT	8	NI	ND	ND			
		P250S	8	0.12	ND	ND			
		I283R	64	0.5	25	ND			
		P295R	32	0.5	ND	ND			
		P295L + Q1074Stop	16	0.25	ND	ND			
		R479K	128	2	1	ND			
		G583R	>64	2	ND	ND			
		A619V	4–8	NI	ND	ND			
		L779F	32	NI	ND	ND			
		A854V	64	1	0.5	ND			
		A859T	8	NI	ND	ND			
		W872C	32	0.12	ND	ND			
		K873N	64	2	ND	ND			
		L926Stop	32	0.5	ND	ND			
		G927D	16	0.25	ND	ND			
		L986P	32	0.5			Yes	ITZ	
		S1081P	8	0.25	ND	ND			
	Tac1p	A21V	8–32	0.06–1	ND	ND			
		G490R + S760R + A761G	8	0.12	ND	ND			
		D603V + P803L	8	0.12	ND	ND			
		G650E	1278–256	4	0.5	ND			
		N900D	8	0.12	ND	ND			
		Q965K + M966V	32	0.5	ND	ND			
		L978W	128	8	0.5	ND			
	Upc2p	P45H	8–>32	0.12–1	ND	ND			
		Q371H	16	0.5	ND	ND			
*C. auris*	Erg11p	F126T	NI	NI	NI	NI			[130,131,133,172,173]
		Y132F	1–256	0.06–16	0.03–0.8	0.015–8			
		K143R	4–256	0.03–16	0.03–0.5	0.015–0.25			
		K143F	64	0.5	0.5	0.25			

**Table 3:** Mutations involved in azole resistance in *A. terreus* and *A. fumigatus* and their in vitro and in vivo impacts. The frequencies mentioned do not necessarily consider all studies published and might be a rough approximation of the actual frequencies.

Species	Protein	Mutation	MIC (µg/mL)			Therapeutic Failure	References
			VRZ	ITZ	PSZ	VRZ	
*A fumigatus*	Cyp51A	G54R	0.125–0.5	2–8	2	G54	[209,211,225,226,227,228,229,230,231,232,233,234,235,236]
		G54E	0.125–0.25	2–16	0.25–2		
		G54W	0.125–0.25	1–16	>16		
		G54V	1	>8			
		G138C	8	>16	>16	ND	
		P216L	1	8→16	1->16	ND	
		M220R	2	>16	2	M220	
		M220I	1	>16	0.5		
		M220V	1–4	8→32	0.5–2		
		M220K	2	16	>16		
		M220L	0.5	>8	>8		
		M220T	0.5–2	32	0.06–0.25		
		G448S	4–8	0.5–8	0.125–1	TR34/L98H	
		TR34/L98H	4–8	>32	0.5–1		
		TR34/L98H/S297T/F497I	0.5	>16	1	ND	
		TR46/Y121F/T289A	>16	>16	2	TR46/Y121F/T289A	
		TR53	16	>16	0.25	ND	
	HapE	P88L	1	>16	0.125	P88L	[217]
*A. terreus*	Cyp51A	M220L	<1	<1	>0.25	Not evaluated	[224]
		M217T	<1	<1	>0.25	Not evaluated	

## References

[1] Hallen-Adams H.E., Suhr M.J. (2017). Fungi in the healthy human gastrointestinal tract. Virulence.

[2] Zhai B., Ola M., Rolling T., Tosini N.L., Joshowitz S., Littmann E.R., Amoretti L.A., Fontana E., Wright R.J., Miranda E. (2020). High-resolution mycobiota analysis reveals dynamic intestinal translocation preceding invasive candidiasis. Nat. Med..

[3] Brown G.D., Denning D.W., Gow N.A.R., Levitz S.M., Netea M.G., White T.C. (2012). Hidden killers: Human fungal infections. Sci. Transl. Med..

[4] Garcia-Rubio R., Cuenca-Estrella M., Mellado E. (2017). Triazole resistance in Aspergillus species: An emerging problem. Drugs.

[5] Lamoth F., Lockhart S.R., Berkow E.L., Calandra T. (2018). Changes in the epidemiological landscape of invasive candidiasis. J. Antimicrob. Chemother..

[6] Moran C., Grussemeyer C.A., Spalding J.R., Benjamin D.K.J., Reed S.D. (2010). Comparison of costs, length of stay, and mortality associated with Candida glabrata and Candida albicans bloodstream infections. Am. J. Infect. Control..

[7] Kontoyiannis D.P., Vaziri I., Hanna H.A., Boktour M., Thornby J., Hachem R., Bodey G.P., Raad I.I. (2001). Risk factors for Candida tropicalis fungemia in patients with cancer. Clin. Infect. Dis..

[8] Patterson T.F., Thompson G.R., Denning D.W., Fishman J.A., Hadley S., Herbrecht R., Kontoyiannis D.P., Marr K.A., Morrison V.A., Nguyen M.H. (2016). Practice Guidelines for the Diagnosis and Management of Aspergillosis: 2016 Update by the Infectious Diseases Society of America. Clin. Infect. Dis..

[9] Pappas P.G., Kauffman C.A., Andes D.R., Clancy C.J., Marr K.A., Ostrosky-Zeichner L., Reboli A.C., Schuster M.G., Vazquez J.A., Walsh T.G. (2016). Clinical Practice Guideline for the Management of Candidiasis: 2016 Update by the Infectious Diseases Society of America. Clin. Infect. Dis..

[10] Cowen L.E., Sanglard D., Howard S.J., Rogers P.D., Perlin D.S. (2014). Mechanisms of antifungal drug resistance. Cold Spring Harb. Perspect. Med..

[11] Berman J., Krysan D.J. (2020). Drug resistance and tolerance in fungi. Nat. Rev. Microbiol..

[12] Jamiu A.T., Albertyn J., Sebolai O.M., Pohl C.H. (2020). Update on Candida krusei, a potential multidrug-resistant pathogen. Med. Mycol..

[13] Araújo D., Henriques M., Silva S. (2017). Portrait of Candida species biofilm regulatory network genes. Trends Microbiol..

[14] Cavalheiro M., Teixeira M.C. (2018). Candida biofilms: Threats, challenges, and promising strategies. Front. Med..

[15] Perry A.M., Hernday A.D., Nobile C.J. (2020). Unraveling how Candida albicans forms sexual biofilms. J. Fungi.

[16] Rodrigues C.F., Rodrigues M.E., Silva S., Henriques M. (2017). Candida glabrata biofilms: How far have we come?. J. Fungi.

[17] Uppuluri P., Lopez Ribot J.L., Prasad R. (2017). Candida albicans biofilms. Candida Albicans: Cellular and Molecular Biology.

[18] Gabaldón T., Naranjo-Ortíz M.A., Marcet-Houben M. (2016). Evolutionary genomics of yeast pathogens in the Saccharomycotina. FEMS Yeast Res..

[19] Mixão V., Gabaldón T. (2018). Hybridization and emergence of virulence in opportunistic human yeast pathogens. Yeast.

[20] Muñoz J.F., Gade L., Chow N.A., Loparev V.N., Juieng P., Berkow E.L., Farrer R.A., Litvintseva A.P., Cuomo C.A. (2018). Genomic insights into multidrug-resistance, mating and virulence in Candida auris and related emerging species. Nat. Commun..

[21] Ksiezopolska E., Gabaldón T. (2018). Evolutionary emergence of drug resistance in Candida opportunistic pathogens. Genes.

[22] Stavrou A.A., Lackner M., Lass-Flörl C., Boekhout T. (2019). The changing spectrum of Saccharomycotina yeasts causing candidemia: Phylogeny mirrors antifungal susceptibility patterns for azole drugs and amphothericin B. FEMS Yeast Res..

[23] Samson R.A., Visagie C.M., Houbraken J., Hong S.B., Hubka V., Klaassen C.H.W., Perrone G., Seifert K.A., Susca A., Tanney J.B. (2014). Phylogeny, identification and nomenclature of the genus Aspergillus. Stud. Mycol..

[24] Frisvad J.C., Larsen T.O. (2016). Extrolites of Aspergillus fumigatus and other pathogenic species in Aspergillus section Fumigati. Front. Microbiol..

[25] Yaguchi T., Horie Y., Tanaka R., Matsuzawa T., Ito J., Nishimura K. (2007). Molecular phylogenetics of multiple genes on Aspergillus section Fumigati isolated from clinical specimens in Japan. Jpn. J. Med. Mycol..

[26] Lass-Flörl C. (2018). Treatment of infections due to Aspergillus terreus species complex. J. Fungi.

[27] Samson R.A., Peterson S.W., Frisvad J.C., Varga J. (2011). New species in Aspergillus section Terrei. Stud. Mycol..

[28] Lackner M., Obermair J., Naschberger V., Raschbichler L.M., Kandelbauer C., Pallua J., Metzlaff J., Furxer S., Lass-Flörl C., Binder U. (2019). Cryptic species of Aspergillus section Terrei display essential physiological features to cause infection and are similar in their virulence potential in Galleria mellonella. Virulence.

[29] Lafayette S.L., Collins C., Zaas A.K., Schell W.A., Betancourt-Quiroz M., Leslie Gunatilaka A.A., Perfect J.R., Cowen L.E. (2010). PKC signaling regulates drug resistance of the fungal pathogen Candida albicans via circuitry comprised of mkc1, calcineurin, and hsp90. PLoS Pathog..

[30] Park H.S., Lee S.C., Cardenas M.E., Heitman J. (2019). Calcium-calmodulin-calcineurin signaling: A globally conserved virulence cascade in eukaryotic microbial pathogens. Cell Host Microbe.

[31] Cowen L.E., Lindquist S. (2005). Hsp90 potentiates the rapid evolution of new traits: Drug resistance in diverse fungi. Science.

[32] Healey K.R., Perlin D.S. (2018). Fungal resistance to echinocandins and the MDR phenomenon in Candida glabrata. J. Fungi.

[33] Dos Reis T.F., Silva L.P., de Castro P.A., do Carmo R.A., Marini M.M., da Silveira J.F., Ferreira B.H., Rodrigues F., Lind A.L., Rokas A. (2019). The Aspergillus fumigatus mismatch repair MSH2 homolog is important for virulence and azole resistance. mSphere.

[34] Boyce K.J., Idnurm A. (2019). Lighting up mutation: A new unbiased system for the measurement of microbial mutation rates. mBio.

[35] Bordallo-Cardona M.Á., Agnelli C., Gómez-Nuñez A., Sánchez-Carrillo C., Bouza E., Muñoz P., Escribano P., Guinea J. (2018). MSH2 gene point mutations are not antifungal resistance markers in Candida glabrata. Antimicrob. Agents Chemother..

[36] Singh S.D., Robbins N., Zaas A.K., Schell W.A., Perfect J.R., Cowen L.E. (2009). Hsp90 governs echinocandin resistance in the pathogenic yeast Candida albicans via calcineurin. PLoS Pathog..

[37] Walker L.A., Munro C.A., De Bruijn I., Lenardon M.D., McKinnon A., Gow N.A.R. (2008). Stimulation of chitin synthesis rescues Candida albicans from echinocandins. PLoS Pathog..

[38] Moreno-Velásquez S.D., Seidel C., Juvvadi P.R., Steinbach W.J., Read N.D. (2017). Caspofungin-Mediated Growth Inhibition and Paradoxical Growth in Aspergillus fumigatus Involve Fungicidal Hyphal Tip Lysis Coupled with Regenerative Intrahyphal Growth and Dynamic Changes in β-1,3-Glucan Synthase Localization. Antimicrob. Agents Chemother..

[39] Valero C., Colabardini A.C., Chiaratto J., Pardeshi L., Alves de Castro P., Filho J.A.F., Silva L.P., Rocha M.C., Malavazi I., Costa J.H. (2020). Aspergillus fumigatus Transcription Factors Involved in the Caspofungin Paradoxical Effect. mBio.

[40] Robbins N., Uppuluri P., Nett J., Rajendran R., Ramage G., Lopez-Ribot J.L., Andes D.A., Cowen L.E. (2011). Hsp90 governs dispersion and drug resistance of fungal biofilms. PLoS Pathog..

[41] Astvad K.M.T., Sanglard D., Delarze E., Hare R.K., Arendrup M.C. (2018). Implications of the EUCAST trailing phenomenon in Candida tropicalis for the in vivo susceptibility in invertebrate and murine models. Antimicrob. Agents Chemother..

[42] Piper P.W., Panaretou B., Millson S.H., Truman A., Mollapour M., Pearl L.H., Prodromou C. (2003). Yeast is selectively hypersensitised to heat shock protein 90 (Hsp90)-targetting drugs with heterologous expression of the human Hsp90β, a property that can be exploited in screens for new Hsp90 chaperone inhibitors. Gene.

[43] Cowen L.E., Singh S.D., Köhler J.R., Collins C., Zaas A.K., Schell W.A., Aziz H., Mylonakis E., Perfect J.R., Whitesell L. (2009). Harnessing Hsp90 function as a powerful, broadly effective therapeutic strategy for fungal infectious disease. Proc. Natl. Acad. Sci. USA.

[44] Ceballos-Garzon A., Amado D., Robert E., Parra Giraldo C.M., Le Pape P. (2020). Impact of calmodulin inhibition by fluphenazine on susceptibility, biofilm formation and pathogenicity of caspofungin-resistant Candida glabrata. J. Antimicrob. Chemother..

[45] Hill J.A., O’Meara T.R., Cowen L.E. (2015). Fitness trade-offs associated with the evolution of resistance to antifungal drug combinations. Cell Rep..

[46] Whitesell L., Robbins N., Huang D.S., McLellan C.A., Shekhar-Guturja T., LeBlanc E.V., Nation C.S., Hui R., Hutchinson A., Collins C. (2019). Structural basis for species-selective targeting of Hsp90 in a pathogenic fungus. Nat. Commun..

[47] Geddes-McAlister J., Shapiro R.S. (2019). New pathogens, new tricks: Emerging, drug-resistant fungal pathogens and future prospects for antifungal therapeutics. Ann. N. Y. Acad. Sci..

[48] Gaur M., Puri N., Manoharlal R., Rai V., Mukhopadhayay G., Choudhury D., Prasad R. (2008). MFS transportome of the human pathogenic yeast Candida albicans. BMC Genomics.

[49] Prasad R., Goffeau A. (2012). Yeast ATP-binding cassette transporters conferring multidrug resistance. Annu. Rev. Microbiol..

[50] Yang H., Tong J., Lee C.W., Ha S., Eom S.H., Im Y.J. (2015). Structural mechanism of ergosterol regulation by fungal sterol transcription factor Upc2. Nat. Commun..

[51] Sagatova A.A., Keniya M.V., Wilson R.K., Monk B.C., Tyndall J.D.A. (2015). Structural insights into binding of the antifungal drug fluconazole to Saccharomyces cerevisiae lanosterol 14α-demethylase. Antimicrob. Agents Chemother..

[52] Perlin D.S. (2015). Echinocandin resistance in Candida. Clin. Infect. Dis..

[53] Arendrup M.C., Perlin D.S. (2014). Echinocandin resistance: An emerging clinical problem?. Curr. Opin. Infect. Dis..

[54] Hou X., Healey K.R., Shor E., Kordalewska M., Ortigosa C.J., Paderu P., Xiao M., Wang H., Zhao Y., Lin L.Y. (2019). Novel FKS1 and FKS2 modifications in a high-level echinocandin resistant clinical isolate of Candida glabrata. Emerg. Microbes Infect..

[55] Ahmad S., Joseph L., Parker J.E., Asadzadeh M., Kelly S.L., Meis J.F., Khan Z. (2019). ERG6 and ERG2 are major targets conferring reduced susceptibility to amphotericin B in clinical Candida glabrata isolates in Kuwait. Antimicrob. Agents Chemother..

[56] Hull C.M., Bader O., Parker J.E., Weig M., Gross U., Warrilow A.G.S., Kelly D.E., Kelly S.L. (2012). Two clinical isolates of Candida glabrata exhibiting reduced sensitivity to amphotericin B both harbor mutations in ERG2. Antimicrob. Agents Chemother..

[57] Sanglard D., Ischer F., Parkinson T., Falconer D., Bille J. (2003). Candida albicans mutations in the ergosterol biosynthetic pathway and resistance to several antifungal agents. Antimicrob. Agents Chemother..

[58] Duesberg P., Stindl R., Hehlmann R. (2001). Origin of multidrug resistance in cells with and without multidrug resistance genes: Chromosome reassortments catalyzed by aneuploidy. Proc. Natl. Acad. Sci. USA.

[59] Selmecki A.M., Dulmage K., Cowen L.E., Anderson J.B., Berman J. (2009). Acquisition of aneuploidy provides increased fitness during the evolution of antifungal drug resistance. PLoS Genet..

[60] Healey K.R., Ortigosa C.J., Shor E., Perlin D.S. (2016). Genetic drivers of multidrug resistance in Candida glabrata. Front. Microbiol..

[61] Bader O., Schwarz A., Kraneveld E.A., Tangwattanchuleeporn M., Schmidt P., Jacobsen M.D., Groβ U., de Groot P.W.J., Weig M. (2012). Gross karyotypic and phenotypic alterations among different progenies of the Candida glabrata CBS138/ATCC2001 reference strain. PLoS ONE.

[62] Carreté L., Ksiezopolska E., Gómez-Molero E., Angoulvant A., Bader O., Fairhead C., Gabaldón T. (2019). Genome comparisons of Candida glabrata serial clinical isolates reveal patterns of genetic variation in infecting clonal populations. Front. Microbiol..

[63] Carreté L., Ksiezopolska E., Pegueroles C., Gómez-Molero E., Saus E., Iraola-Guzmán S., Loska D., Bader O., Fairhead C., Gabaldón T. (2018). Patterns of genomic variation in the opportunistic pathogen Candida glabrata suggest the existence of mating and a secondary association with humans. Curr. Biol..

[64] Morio F., Jensen R.H., Le Pape P., Arendrup M.C. (2017). Molecular basis of antifungal drug resistance in yeasts. Int. J. Antimicrob. Agents.

[65] Ben-Ami R., Garcia-Effron G., Lewis R.E., Gamarra S., Leventakos K., Perlin D.S., Kontoyiannis D.P. (2011). Fitness and virulence costs of Candida albicans FKS1 hot spot mutations associated with echinocandin resistance. J. Infect. Dis..

[66] Vale-Silva L.A., Sanglard D. (2015). Tipping the balance both ways: Drug resistance and virulence in Candida glabrata. FEMS Yeast Res..

[67] Cheng S., Clancy C.J., Hartman D.J., Hao B., Nguyen M.H. (2014). Candida glabrata intra-abdominal candidiasis is characterized by persistence within the peritoneal cavity and abscesses. Infect. Immun..

[68] Howard S.J., Livermore J., Sharp A., Goodwin J., Gregson L., Alastruey-Izquierdo A., Perlin D.S., Warn P.A., Hope W.W. (2011). Pharmacodynamics of echinocandins against Candida glabrata: Requirement for dosage escalation to achieve maximal antifungal activity in neutropenic hosts. Antimicrob. Agents Chemother..

[69] Healey K.R., Nagasaki Y., Zimmerman M., Kordalewska M., Park S., Zhao Y., Perlin D.S. (2017). The gastrointestinal tract is a major source of echinocandin drug resistance in a murine model of Candida glabrata colonization and systemic dissemination. Antimicrob. Agents Chemother..

[70] Costa-de-Oliveira S., Rodrigues A.G. (2020). Candida albicans Antifungal Resistance and Tolerance in Bloodstream Infections: The Triad Yeast-Host-Antifungal. Microorganisms.

[71] Zhao Y., Prideaux B., Nagasaki Y., Lee M.H., Chen P.Y., Blanc L., Ho H., Clancy C.J., Nguyen M.H., Dartois V. (2017). Unraveling drug penetration of echinocandin antifungals at the site of infection in an intra-abdominal abscess model. Antimicrob. Agents Chemother..

[72] Lockhart S.R., Iqbal N., Cleveland A.A., Farley M.M., Harrison L.H., Bolden C.B., Baughman W., Stein B., Hollick R., Park B.J. (2012). Species identification and antifungal susceptibility testing of Candida bloodstream isolates from population-based surveillance studies in two U.S. cities from 2008 to 2011. J. Clin. Microbiol..

[73] Remington T.L., Isaac A., Vickers D.M., Fuller J., Wrenn Smith S. (2018). Epidemiology of candidemia at a tertiary Canadian hospital, 2004–2013. Can. J. Infect. Dis. Med. Microbiol..

[74] Chapman B., Slavin M., Marriott D., Halliday C., Kidd S., Arthur I., Bak N., Heath C.H., Kennedy K., Morrissey C.O. (2017). Australian and New Zealand Mycoses Interest Group. Changing epidemiology of candidaemia in Australia. J. Antimicrob. Chemother..

[75] Ala-Houhala M., Valkonen M., Kolho E., Friberg N., Anttila V.J. (2019). Clinical and microbiological factors associated with mortality in candidemia in adult patients 2007–2016. Infect. Dis..

[76] Astvad K.M.T., Johansen H.K., Røder B.L., Rosenvinge F.S., Knudsen J.D., Lemming L., Schønheyder H.C., Hare R.K., Kristensen L., Arendrup M.C. (2018). Update from a 12-year nationwide fungemia surveillance: Increasing intrinsic and acquired resistance causes concern. J. Clin. Microbiol..

[77] Hesstvedt L., Gaustad P., Andersen C.T., Haarr E., Hannula R., Haukland H.H., Hermansen N.O., Larssen K.W., Mylvaganam H., Ranheim T.E. (2015). Twenty-two years of candidaemia surveillance: Results from a Norwegian national study. Clin. Microbiol. Infect..

[78] Lindberg E., Hammarström H., Ataollahy N., Kondori N. (2019). Species distribution and antifungal drug susceptibilities of yeasts isolated from the blood samples of patients with candidemia. Sci. Rep..

[79] Pfaller M.A., Diekema D.J., Turnidge J.D., Castanheira M., Jones R.N. (2019). Twenty years of the SENTRY Antifungal Surveillance Program: Results for Candida species from 1997–2016. Open Forum Infect. Dis..

[80] Poikonen E., Lyytikäinen O., Anttila V.J., Koivula I., Lumio J., Kotilainen P., Syrjälä H., Ruutu P. (2010). Secular trend in candidemia and the use of fluconazole in Finland, 2004–2007. BMC Infect. Dis..

[81] Alobaid K., Khan Z. (2019). Epidemiologic characteristics of adult candidemic patients in a secondary hospital in Kuwait: A retrospective study. J. Mycol. Med..

[82] Hii I.M., Chang H.L., Lin L.C., Lee Y.L., Liu Y.M., Liu C.E., Chen C.H., Cheng Y.R., Chang C.Y. (2015). Changing epidemiology of candidemia in a medical center in middle Taiwan. J. Microbiol. Immunol. Infect..

[83] Kakeya H., Shibata W., Yamada K., Kaneko Y. (2019). National trends in the Japanese distribution of major Candida species causing candidemia during 2003–2017: A report by the Epidemiological Investigation Committee for Human Mycoses in Japan. Open Forum Infect. Dis..

[84] Tortorano A.M., Prigitano A., Lazzarini C., Passera M., Deiana M.L., Cavinato S., De Luca C., Grancini A., Lo Cascio G., Ossi C. (2013). A 1-year prospective survey of candidemia in Italy and changing epidemiology over one decade. Infection.

[85] Lewis J.S., Wiederhold N.P., Wickes B.L., Patterson T.F., Jorgensen J.H. (2013). Rapid emergence of echinocandin resistance in Candida glabrata resulting in clinical and microbiologic failure. Antimicrob. Agents Chemother..

[86] Shields R.K., Nguyen M.H., Press E.G., Kwa A.L., Cheng S., Du C., Clancy C.J. (2012). The presence of an FKS mutation rather than MIC is an independent risk factor for failure of echinocandin therapy among patients with invasive candidiasis due to Candida glabrata. Antimicrob. Agents Chemother..

[87] Chapeland-Leclerc F., Hennequin C., Papon N., Noël T., Girard A., Socié G., Ribaud P., Lacroix C. (2010). Acquisition of flucytosine, azole, and caspofungin resistance in Candida glabrata bloodstream isolates serially obtained from a hematopoietic stem cell transplant recipient. Antimicrob. Agents Chemother..

[88] Cleary J.D., Garcia-Effron G., Chapman S.W., Perlin D.S. (2008). Reduced Candida glabrata susceptibility secondary to an FKS1 mutation developed during candidemia treatment. Antimicrob. Agents Chemother..

[89] Costa de Oliveira S., Miranda I.M., Silva R.M., Silva A.P.E., Rocha R., Amorim A., Rodrigues A.G., Pina-Vaz C. (2011). FKS2 mutations associated with decreased echinocandin susceptibility of Candida glabrata following anidulafungin therapy. Antimicrob. Agents Chemother..

[90] Garcia-Effron G., Chua D.J., Tomada J.R., Di Persio J., Perlin D.S., Ghannoum M., Bonilla H. (2010). Novel FKS mutations associated with echinocandin resistance in Candida species. Antimicrob. Agents Chemother..

[91] Grosset M., Desnos-Ollivier M., Godet C., Kauffmann-Lacroix C., Cazenave-Roblot F. (2016). Recurrent episodes of candidemia due to Candida glabrata, Candida tropicalis and Candida albicans with acquired echinocandin resistance. Med. Mycol. Case Rep..

[92] Pfeiffer C.D., Garcia-Effron G., Zaas A.K., Perfect J.R., Perlin D.S., Alexander B.D. (2010). Breakthrough invasive candidiasis in patients on micafungin. J. Clin. Microbiol..

[93] Thompson G.R., Wiederhold N.P., Vallor A.C., Villareal N.C., Lewis J.S., Patterson T.F. (2008). Development of caspofungin resistance following prolonged therapy for invasive candidiasis secondary to Candida glabrata infection. Antimicrob. Agents Chemother..

[94] Wright W.F., Bejou N., Shields R.K., Marr K., McCarty T.P., Pappas P.G. (2019). Amphotericin B induction with voriconazole consolidation as salvage therapy for FKS-associated echinocandin resistance in Candida glabrata septic arthritis and osteomyelitis. Antimicrob. Agents Chemother..

[95] Xiao M., Fan X., Hou X., Chen S.C.A., Wang H., Kong F., Sun Z.Y., Chu Y.Z., Xu Y.C. (2018). Clinical characteristics of the first cases of invasive candidiasis in China due to pan-echinocandin-resistant Candida tropicalis and Candida glabrata isolates with delineation of their resistance mechanisms. Infect. Drug Resist..

[96] Alexander B.D., Johnson M.D., Pfeiffer C.D., Jiménez-Ortigosa C., Catania J., Booker R., Castanheira M., Messer S.A., Perlin D.S., Pfaller M.A. (2013). Increasing echinocandin resistance in Candida glabrata: Clinical failure correlates with presence of FKS mutations and elevated minimum inhibitory concentrations. Clin. Infect. Dis..

[97] Hull C.M., Parker J.E., Bader O., Weig M., Gross U., Warrilow A.G.S., Kelly D.E., Kelly S.L. (2012). Facultative sterol uptake in an ergosterol-deficient clinical isolate of Candida glabrata harboring a missense mutation in ERG11 and exhibiting cross-resistance to azoles and amphotericin B. Antimicrob. Agents Chemother..

[98] Ferrari S., Ischer F., Calabrese D., Posteraro B., Sanguinetti M., Fadda G., Rohde B., Bauser C., Bader O., Sanglard D. (2009). Gain of function mutations in CgPDR1 of Candida glabrata not only mediate antifungal resistance but also enhance virulence. PLoS Pathog..

[99] Tian Y., Gao N., Ni Q., Mao Y., Dong D., Huang X., Jiang C., Li Z., Zhang L., Wang X. (2018). Sequence modification of the master regulator Pdr1 interferes with its transcriptional autoregulation and confers altered azole resistance in Candida glabrata. FEMS Yeast Res..

[100] Usher J., Haynes K. (2019). Attenuating the emergence of anti-fungal drug resistance by harnessing synthetic lethal interactions in a model organism. PLoS Genet..

[101] Thakur J.K., Arthanari H., Yang F., Pan S.J., Fan X., Breger J., Frueh D.P., Gulshan K., Li D.K., Mylonakis E. (2008). A nuclear receptor-like pathway regulating multidrug resistance in fungi. Nature.

[102] Vale-Silva L., Ischer F., Leibundgut-Landmann S., Sanglard D. (2013). Gain-of-function mutations in PDR1, a regulator of antifungal drug resistance in Candida glabrata, control adherence to host cells. Infect. Immun..

[103] Noble J.A., Tsai H.F., Suffis S.D., Su Q., Myers T.G., Bennett J.E. (2013). STB5 is a negative regulator of azole resistance in Candida glabrata. Antimicrob. Agents Chemother..

[104] Nishikawa J.L., Boeszoermenyi A., Vale-Silva L.A., Torelli R., Posteraro B., Sohn Y.J., Ji F., Gelev V., Sanglard D., Sanguinetti M. (2016). Inhibiting fungal multidrug resistance by disrupting an activator–mediator interaction. Nature.

[105] Vu B.G., Thomas G.H., Moye-Rowley W.S. (2019). Evidence that ergosterol biosynthesis modulates activity of the Pdr1 transcription factor in Candida glabrata. mBio.

[106] Whaley S.G., Caudle K.E., Vermitsky J.P., Chadwick S.G., Toner G., Barker K.S., Gygax S.E., Rogers P.D. (2014). UPC2A is required for high-level azole antifungal resistance in Candida glabrata. Antimicrob. Agents Chemother..

[107] Arastehfar A., Daneshnia F., Salehi M., Yaşar M., Hoşbul T., Ilkit M., Pan W., Hagen F., Arslan N., Türk-Dağı H. (2020). Low level of antifungal resistance of Candida glabrata blood isolates in Turkey: Fluconazole minimum inhibitory concentration and FKS mutations can predict therapeutic failure. Mycoses.

[108] Dannaoui E., Desnos-Ollivier M., Garcia-Hermoso D., Grenouillet F., Cassaing S., Baixench M.T., Bretagne S., Dromer F., Lortholary O., French Mycoses Study Group (2012). Candida spp. with acquired echinocandin resistance, France, 2004–2010. Emerg. Infect. Dis..

[109] Sasso M., Roger C., Lachaud L. (2017). Rapid emergence of FKS mutations in Candida glabrata isolates in a peritoneal candidiasis. Med. Mycol. Case Rep..

[110] Biswas C., Marcelino V.R., van Hal S., Halliday C., Martinez E., Wang Q., Kidd S., Kennedy K., Marriott D., Morrissey C.O. (2018). Whole genome sequencing of Australian Candida glabrata isolates reveals genetic diversity and novel sequence types. Front. Microbiol..

[111] Fraser M., Borman A.M., Thorn R., Lawrance L.M. (2020). Resistance to echinocandin antifungal agents in the United Kingdom in clinical isolates of Candida glabrata: Fifteen years of interpretation and assessment. Med. Mycol..

[112] Klotz U., Schmidt D., Willinger B., Steinmann E., Buer J., Rath P.M., Steinmann J. (2016). Echinocandin resistance and population structure of invasive Candida glabrata isolates from two University hospitals in Germany and Austria. Mycoses.

[113] Rivero-Menendez O., Navarro-Rodriguez P., Bernal-Martinez L., Martin-Cano G., Lopez-Perez L., Sanchez-Romero I., Perez-Ayala A., Capilla J., Zaragoza O., Alastruey-Izquierdo A. (2019). Clinical and laboratory development of echinocandin resistance in Candida glabrata: Molecular characterization. Front. Microbiol..

[114] Kritikos A., Neofytos D., Khanna N., Schreiber P.W., Boggian K., Bille J., Schrenzel J., Mühlethaler K., Zbinden R., Bruderer T. (2018). Fungal Infection Network of Switzerland (FUNGINOS). Accuracy of Sensititre YeastOne echinocandins epidemiological cut-off values for identification of FKS mutant Candida albicans and Candida glabrata: A ten year national survey of the Fungal Infection Network of Switzerland (FUNGINOS). Clin. Microbiol. Infect..

[115] Prigent G., Aït-Ammar N., Levesque E., Fekkar A., Costa J., El Anbassi S., Foulet F., Duvoux C., Merle J.C., Dannaoui E. (2017). Echinocandin resistance in Candida species isolates from liver transplant recipients. Antimicrob. Agents Chemother..

[116] Zimbeck A.J., Iqbal N., Ahlquist A.M., Farley M.M., Harrison L.H., Chiller T., Lockhart S.R. (2010). FKS mutations and elevated echinocandin MIC values among Candida glabrata isolates from U.S. population-based surveillance. Antimicrob. Agents Chemother..

[117] Barber A.E., Weber M., Kaerger K., Duerschmied D., Markert A., Guthke R., Walther G., Kurzai O. (2019). Comparative genomics of serial Candida glabrata isolates and the rapid acquisition of echinocandin resistance during therapy. Antimicrob. Agents Chemother..

[118] Naicker S.D., Magobo R.E., Zulu T.G., Maphanga T.G., Luthuli N., Lowman W., Govender N.P. (2016). Two echinocandin-resistant Candida glabrata FKS mutants from South Africa. Med. Mycol. Case Rep..

[119] Spettel K., Barousch W., Makristathis A., Zeller I., Nehr M., Selitsch B., Lackner M., Rath P.M., Steinmann J., Willinger B. (2019). Analysis of antifungal resistance genes in Candida albicans and Candida glabrata using next generation sequencing. PLoS ONE.

[120] Pham C.D., Iqbal N., Bolden C.B., Kuykendall R.J., Harrison L.H., Farley M.M., Schaffner W., Beldavs Z.G., Chiller T.M., Park B.J. (2014). Role of FKS mutations in Candida glabrata: MIC values, echinocandin resistance, and multidrug resistance. Antimicrob. Agents Chemother..

[121] Goemaere B., Lagrou K., Spriet I., Hendrickx M., Becker B. (2018). Clonal spread of Candida glabrata bloodstream isolates and fluconazole resistance affected by prolonged exposure: A 12-year single-center study in Belgium. Antimicrob. Agents Chemother..

[122] Beyda N.D., John J., Kilic A., Alam M.J., Lasco T.M., Garey K.W. (2014). 2014. FKS mutant Candida glabrata: Risk factors and outcomes in patients with candidemia. Clin. Infect. Dis..

[123] Biswas C., Chen S.C.A., Halliday C., Kennedy K., Playford E.G., Marriott D.J., Slavin M.A., Sorrell T.C., Sintchenko V. (2017). Identification of genetic markers of resistance to echinocandins, azoles and 5-fluorocytosine in Candida glabrata by next-generation sequencing: A feasibility study. Clin. Microbiol. Infect..

[124] Hou X., Xiao M., Wang H., Yu S., Zhang G., Zhao Y., Xu Y. (2018). Profiling of PDR1 and MSH2 in Candida glabrata bloodstream isolates from a multicenter study in China. Antimicrob. Agents Chemother..

[125] Garcia-Effron G., Kontoyiannis D.P., Lewis R.E., Perlin D.S. (2008). Caspofungin-resistant Candida tropicalis strains causing breakthrough fungemia in patients at high risk for hematologic malignancies. Antimicrob. Agents Chemother..

[126] Jensen R.H., Johansen H.K., Arendrup M.C. (2013). Stepwise development of a homozygous S80P substitution in Fks1p, conferring echinocandin resistance in Candida tropicalis. Antimicrob. Agents Chemother..

[127] Marcos-Zambrano L.J., Escribano P., Sánchez C., Muñoz P., Bouza E., Guinea J. (2014). Antifungal resistance to fluconazole and echinocandins is not emerging in yeast isolates causing fungemia in a Spanish tertiary care center. Antimicrob. Agents Chemother..

[128] Desnos-Ollivier M., Bretagne S., Raoux D., Hoinard D., Dromer F., Dannaoui E., European Committee on Antibiotic Susceptibility Testing (2008). Mutations in the fks1 gene in Candida albicans, C. tropicalis, and C. krusei correlate with elevated caspofungin MICs uncovered in AM3 medium using the method of the European Committee on Antibiotic Susceptibility testing. Antimicrob. Agents Chemother..

[129] Arastehfar A., Daneshnia F., Hilmioğlu-Polat S., Ilkit M., Yaşar M., Polat F., Metin D.Y., Dökümcü Ü.Z., Pan W., Hagen F. (2020). Genetically-related micafungin-resistant C. parapsilosis blood isolates harboring a novel mutation R658G in hotspot1-Fks1p: A new challenge?. J. Antimicrob. Chemother..

[130] Rhodes J., Abdolrasouli A., Farrer R.A., Cuomo C.A., Aanensen D.M., Armstrong-James D., Fisher M.C., Schelenz S. (2018). Genomic epidemiology of the UK outbreak of the emerging human fungal pathogen Candida auris. Emerg. Microbes Infect..

[131] Chowdhary A., Prakash A., Sharma C., Kordalewska M., Kumar A., Sarma S., Tarai B., Singh A., Upadhyaya G., Upadhyay S. (2018). A multicentre study of antifungal susceptibility patterns among 350 Candida auris isolates (2009–17) in India: Role of the ERG11 and FKS1 genes in azole and echinocandin resistance. J. Antimicrob. Chemother..

[132] Kordalewska M., Lee A., Park S., Berrio I., Chowdhary A., Zhao Y., Perlin D.S. (2018). Understanding echinocandin resistance in the emerging pathogen Candida auris. Antimicrob. Agents Chemother..

[133] Ahmad S., Khan Z., Al-Sweih N., Alfouzan W., Joseph L. (2020). Candida auris in various hospitals across Kuwait and their susceptibility and molecular basis of resistance to antifungal drugs. Mycoses.

[134] Berkow E.L., Lockhart S.R. (2018). Activity of CD101, a long-acting echinocandin, against clinical isolates of Candida auris. Diagn. Microbiol. Infect. Dis..

[135] Jiménez-Ortigosa C., Moore M., Denning D.W., Perlin D.S. (2017). Emergence of Echinocandin Resistance Due to a Point Mutation in the fks1 Gene of Aspergillus fumigatus in a Patient with Chronic Pulmonary Aspergillosis. Antimicrob Agents Chemother..

[136] Dellière S., Healey K., Gits-Muselli M., Carrara B., Barbaro A., Guigue N., Lecefel C., Touratier S., Desnos-Ollivier M., Perlin D.S. (2016). Fluconazole and echinocandin resistance of Candida glabrata correlates better with antifungal drug exposure rather than with MSH2 mutator genotype in a French cohort of patients harboring low rates of resistance. Front. Microbiol..

[137] Singh A., Healey K.R., Yadav P., Upadhyaya G., Sachdeva N., Sarma S., Kumar A., Tarai B., Perlin D.S., Chowdhary A. (2018). Absence of azole or echinocandin resistance in Candida glabrata isolates in India despite background prevalence of strains with defects in the DNA mismatch repair pathway. Antimicrob. Agents Chemother..

[138] Chakrabarti A., Sood P., Rudramurthy S.M., Chen S., Kaur H., Capoor M., Chhina D., Rao R., Eshwara V.K., Xes I. (2015). Incidence, characteristics and outcome of ICU-acquired candidemia in India. Intensive Care Med..

[139] Sellami A., Sellami H., Néji S., Makni F., Abbes S., Cheikhrouhou F., Chelly H., Bouaziz M., Hammami B., Ben Jemma M. (2011). Antifungal susceptibility of bloodstream Candida isolates in Sfax Hospital: Tunisia. Mycopathologia.

[140] Megri Y., Arastehfar A., Boekhout T., Daneshnia F., Hörtnagl C., Sartori B., Hafez A., Pan W., Lass-Flörl C., Hamrioui B. (2020). Candida tropicalis is the most prevalent yeast species causing candidemia in Algeria: The urgent need for antifungal stewardship and infection control measures. Antimicrob. Resist. Infect. Control.

[141] Tan T.Y., Hsu L.Y., Alejandria M.M., Chaiwarith R., Chinniah T., Chayakulkeeree M., Choudhury S., Chen Y.H., Shin J.H., Kiratisin P. (2016). Antifungal susceptibility of invasive Candida bloodstream isolates from the Asia-Pacific region. Med. Mycol..

[142] Zuza-Alves D.L., Silva-Rocha W.P., Chaves G.M. (2017). An update on Candida tropicalis based on basic and clinical approaches. Front. Microbiol..

[143] Khan Z., Ahmad S., Mokaddas E., Vayalil S., Meis J.F., Joseph L., Abdullah A. (2018). Development of echinocandin resistance in Candida tropicalis following short-term exposure to caspofungin for empiric therapy. Antimicrob. Agents Chemother..

[144] Ko J.H., Jung D.S., Lee J.Y., Kim H.A., Ryu S.Y., Jung S.I., Joo E.J., Cheon S., Kim Y.S., Kim S.W. (2019). Poor prognosis of Candida tropicalis among non-albicans candidemia: A retrospective multicenter cohort study, Korea. Diagn. Microbiol. Infect. Dis..

[145] Arendrup M.C., Bruun B., Christensen J.J., Fuursted K., Johansen H.K., Kjældgaard P., Knudsen J.D., Kristensen L., Møller J., Nielsen L. (2011). National Surveillance of Fungemia in Denmark (2004 to 2009). J. Clin. Microbiol..

[146] Chen P.Y., Chuang Y.C., Wu U.I., Sun H.Y., Wang J.T., Sheng W.H., Lo H.J., Wang H.Y., Chen Y.C., Chang S.C. (2019). Clonality of fluconazole-nonsusceptible Candida tropicalis in bloodstream infections, Taiwan, 2011–2017. Emerg. Infect. Dis..

[147] Chong Y., Shimoda S., Yakushiji H., Ito Y., Miyamoto T., Shimono N., Kamimura T., Akashi K. (2012). Fatal candidemia caused by azole-resistant Candida tropicalis in patients with hematological malignancies. J. Infect. Chemother..

[148] Fan X., Xiao M., Liao K., Kudinha T., Wang H., Zhang L., Hou X., Kong F., Xu Y.C. (2017). Notable increasing trend in azole non-susceptible Candida tropicalis causing invasive candidiasis in China (August 2009 to July 2014): Molecular epidemiology and clinical azole consumption. Front. Microbiol..

[149] Arastehfar A., Daneshnia F., Hafez A., Khodavaisy S., Najafzadeh M.J., Charsizadeh A., Zarrinfar H., Salehi M., Shahrabadi Z.Z., Sasani E. (2020). Antifungal susceptibility, genotyping, resistance mechanism, and clinical profile of Candida tropicalis blood isolates. Med. Mycol..

[150] Arastehfar A., Hilmioğlu-Polat S., Daneshnia F., Hafez A., Salehi M., Polat F., Yaşar M., Arslan N., Hoşbul T., Ünal N. (2020). Recent increase in the prevalence of fluconazole-non-susceptible Candida tropicalis blood isolates in Turkey: Clinical implication of azole-non-susceptible and fluconazole tolerant phenotypes and genotyping. Front. Microbiol..

[151] Castanheira M., Deshpande L.M., Messer S.A., Rhomberg P.R., Pfaller M.A. (2020). Analysis of global antifungal surveillance results reveals predominance of Erg11 Y132F alteration among azole-resistant Candida parapsilosis and Candida tropicalis and country-specific isolate dissemination. Int. J. Antimicrob. Agents..

[152] Fan X., Xiao M., Zhang D., Huang J.J., Wang H., Hou X., Zhang L., Kong F., Chen S.C.A., Tong Z.H. (2019). Molecular mechanisms of azole resistance in Candida tropicalis isolates causing invasive candidiasis in China. Clin. Microbiol. Infect..

[153] Jiang C., Dong D., Yu B., Cai G., Wang X., Ji Y., Peng Y. (2013). Mechanisms of azole resistance in 52 clinical isolates of Candida tropicalis in China. J. Antimicrob. Chemother..

[154] Vandeputte P., Larcher G., Berges T., Renier G., Chabasse D., Bouchara J.P. (2005). Mechanisms of azole resistance in a clinical isolate of Candida tropicalis. Antimicrob. Agents Chemother..

[155] Jin L., Cao Z., Wang Q., Wang Y., Wang X., Chen H., Wang H. (2018). MDR1 overexpression combined with ERG11 mutations induce high-level fluconazole resistance in Candida tropicalis clinical isolates. BMC Infect. Dis..

[156] Teo J.Q.M., Lee S.J.Y., Tan A.L., Lim R.S.M., Cai Y., Lim T.P., Kwa A.L.H. (2019). Molecular mechanisms of azole resistance in Candida bloodstream isolates. BMC Infect. Dis..

[157] Jiang C., Ni Q., Dong D., Zhang L., Li Z., Tian Y., Peng Y. (2016). The role of UPC2 gene in azole-resistant Candida tropicalis. Mycopathologia.

[158] Scordino F., Giuffre L., Barberi G., Merlo F.M., Orlando M.G., Giosa D., Romeo O. (2018). Multilocus sequence typing reveals a new cluster of closely related Candida tropicalis genotypes in Italian patients with neurological disorders. Front. Microbiol..

[159] Chou H.H., Lo H.J., Chen K.W., Liao M.H., Li S.Y. (2007). Multilocus sequence typing of Candida tropicalis shows clonal cluster enriched in isolates with resistance or trailing growth of fluconazole. Diagn. Microbiol. Infect. Dis..

[160] Chakrabarti A., Chatterjee S.S., Rao K.L.N., Zameer M.M., Shivaprakash M.R., Singhi S., Singh R., Varma S.C. (2009). Recent experience with fungaemia: Change in species distribution and azole resistance. Scand. J. Infect. Dis..

[161] Choi M.J., Won E.J., Shin J.H., Kim S.H., Lee W.G., Kim M.N., Lee K., Shin M.G., Suh S.P., Ryang D.W. (2016). Resistance mechanisms and clinical features of fluconazole-nonsusceptible Candida tropicalis isolates compared with fluconazole-less-susceptible isolates. Antimicrob. Agents Chemother..

[162] Forastiero A., Mesa-Arango A.C., Alastruey-Izquierdo A., Alcazar-Fuoli L., Bernal-Martinez L., Pelaez T., Lopez J.F., Grimalt J.O., Gomez-Lopez A., Cuesta I. (2013). Candida tropicalis antifungal cross-resistance is related to different azole target (Erg11p) modifications. Antimicrob. Agents Chemother..

[163] Tan J., Zhang J., Chen W., Sun Y., Wan Z., Li R., Liu W. (2015). The A395T mutation in ERG11 gene confers fluconazole resistance in Candida tropicalis causing candidemia. Mycopathologia.

[164] Choi Y.J., Kim Y.J., Yong D., Byun J.H., Kim T.S., Chang Y.S., Choi M.J., Byeon S.A., Won E.J., Kim S.H. (2018). Fluconazole-resistant Candida parapsilosis bloodstream isolates with Y132F mutation in ERG11 gene, South Korea. Emerg. Infect. Dis..

[165] Grossman N.T., Pham C.D., Cleveland A.A., Lockhart S.R. (2015). Molecular mechanisms of fluconazole resistance in Candida parapsilosis isolates from a U.S. surveillance system. Antimicrob. Agents Chemother..

[166] Asadzadeh M., Ahmad S., Al-Sweih N., Khan Z. (2017). Epidemiology and molecular basis of resistance to fluconazole among clinical Candida parapsilosis isolates in Kuwait. Microb. Drug Resist..

[167] Souza A.C.R., Fuchs B.B., Pinhati H.M.S., Siqueira R.A., Hagen F., Meis J.F., Mylonakis E., Colombo A.L. (2015). Candida parapsilosis resistance to fluconazole: Molecular mechanisms and in vivo impact in infected Galleria mellonella larvae. Antimicrob. Agents Chemother..

[168] Thomaz D.Y., de Almeida J.N., Lima G.M.E., de Oliveira Nunes M., Camargo C.H., Grenfell R.C., Benard G., Del Negro G.M.B. (2018). An azole-resistant Candida parapsilosis outbreak: Clonal persistence in the intensive care unit of a Brazilian teaching hospital. Front. Microbiol..

[169] Singh A., Singh P.K., de Groot T., Kumar A., Mathur P., Tarai B., Sachdeva N., Upadhyaya G., Sarma S., Meis J.F. (2019). Emergence of clonal fluconazole-resistant Candida parapsilosis clinical isolates in a multicentre laboratory-based surveillance study in India. J. Antimicrob. Chemother..

[170] Arastehfar A., Daneshnia F., Hilmioğlu-Polat S., Fang W., Yaşar M., Polat F., Metin D.Y., Rigole P., Coenye T., Ilkit M. (2020). First report of candidemia clonal outbreak caused by emerging fluconazole-resistant Candida parapsilosis isolates harboring Y132F and/or Y132F+K143R in Turkey. Antimicrob. Agents Chemother..

[171] Zhang L., Xiao M., Watts M.R., Wang H., Fan X., Kong F., Xu Y.C. (2015). Development of fluconazole resistance in a series of Candida parapsilosis isolates from a persistent candidemia patient with prolonged antifungal therapy. BMC Infect. Dis..

[172] Lockhart S.R., Etienne K.A., Vallabhaneni S., Farooqi J., Chowdhary A., Govender N.P., Colombo A.L., Calvo B., Cuomo C.A., Desjardins C.A. (2017). Simultaneous emergence of multidrug-resistant Candida auris on 3 continents confirmed by whole-genome sequencing and epidemiological analyses. Clin. Infect. Dis..

[173] Kwon Y.J., Shin J.H., Byun S.A., Choi M.J., Won E.J., Lee D., Lee S.Y., Chun S., Lee J.H., Choi H.J. (2019). Candida auris clinical isolates from South Korea: Identification, antifungal susceptibility, and genotyping. J. Clin. Microbiol..

[174] Nucci M., Queiroz-Telles F., Alvarado-Matute T., Tiraboschi I.N., Cortes J., Zurita J., Guzman-Blanco M., Santolaya M.E., Thompson L., Sifuentes-Osornio J. (2013). Latin American Invasive Mycosis Network. Epidemiology of candidemia in Latin America: A laboratory based survey. PLoS ONE.

[175] Ko J.H., Jung D.S., Lee J.Y., Kim H.A., Ryu S.Y., Jung S.I., Joo E.J., Cheon S., Kim Y.S., Kim S.W. (2019). Changing epidemiology of non-albicans candidemia in Korea. J. Infect. Chemother..

[176] Won E.J., Shin J.H., Choi M.J., Lee W.G., Park Y.J., Uh Y., Kim S.Y., Lee M.K., Kim S.H., Shin M.G. (2015). Antifungal susceptibilities of bloodstream isolates of Candida species from nine hospitals in Korea: Application of new antifungal breakpoints and relationship to antifungal usage. PLoS ONE.

[177] Guo F., Yang Y., Kang Y., Zang B., Cui W., Qin B., Qin Y., Fang Q., Qin T., Jiang D. (2013). China-SCAN Team. Invasive candidiasis in intensive care units in China: A multicentre prospective observational study. J. Antimicrob. Chemother..

[178] Khan Z., Ahmad S., Al-Sweih N., Mokaddas E., Al-Banwan K., Alfouzan W., Al-Obaid I., Al-Obaid K., Asadzadeh M., Jeragh A. (2019). Changing trends in epidemiology and antifungal susceptibility patterns of six bloodstream Candida species isolates over a 12-year period in Kuwait. PLoS ONE.

[179] Dimopoulos G., Velegraki A., Falagas M.E. (2009). A 10-year survey of antifungal susceptibility of candidemia isolates from intensive care unit patients in Greece. Antimicrob. Agents Chemother..

[180] Puig-Asensio M., Padilla B., Garnacho-Montero J., Zaragoza O., Aguado J.M., Zaragoza R., Montejo M., Muñoz P., Ruiz-Camps I., Cuenca-Estrella M. (2014). CANDIPOP Project; GEIH-GEMICOMED (SEIMC); REIPI. Epidemiology and predictive factors for early and late mortality in Candida bloodstream infections: A population-based surveillance in Spain. Clin. Microbiol. Infect..

[181] Papadimitriou-Olivgeris M., Spiliopoulou A., Kolonitsiou F., Bartzavali C., Lambropoulou A., Xaplanteri P., Anastassiou E.D., Marangos M., Spiliopoulou I., Christofidou M. (2019). Increasing incidence of candidaemia and shifting epidemiology in favor of Candida non-albicans in a 9-year-period (2009–2017) in a university Greek hospital. Infection.

[182] Govender N.P., Patel J., Magobo R.E., Naicker S., Wadula J., Whitelaw A., Coovadia Y., Kularatne R., Govind C., Lockhart S.R. (2016). Emergence of azole-resistant Candida parapsilosis causing bloodstream infection: Results from laboratory-based sentinel surveillance in South Africa. J. Antimicrob. Chemother..

[183] Tóth R., Nosek J., Mora-Montes H.M., Gabaldon T., Bliss J.M., Nosanchuk J.D., Turner S.A., Butler G., Vágvölgyi C., Gácser M. (2019). Candida parapsilosis: From genes to the bedside. Clin. Microbiol. Rev..

[184] Kim J.H., Suh J.W., Kim J.Y., Lee H., Kim S.B., Sohn J.W., Kim M.J. (2019). Epidemiology and antifungal susceptibility of candidemia among adult patients at a tertiary care hospital in South Korea during an 8-year period. Open Forum Infect. Dis..

[185] Berkow E.L., Manigaba K., Parker J.E., Barker K.S., Kelly S.L., Rogers P.D. (2015). Multidrug transporters and alterations in sterol biosynthesis contribute to azole antifungal resistance in Candida parapsilosis. Antimicrob. Agents Chemother..

[186] Arastehfar A., Daneshnia F., Najafzadeh M.J., Hagen F., Mahmoudi S., Salehi M., Zarrinfar H., Namvar Z., Shahrabadi Z.Z., Khodavaisy S. (2020). Evaluation of molecular epidemiology, clinical characteristics, antifungal susceptibility profiles, and molecular mechanisms of antifungal resistance of Iranian Candida parapsilosis species complex blood isolates. Front. Cell. Infect. Microbiol..

[187] Branco J., Silva A.P., Silva R.M., Silva-Dias A., Pina-Vaz C., Butler G., Rodrigues A.G., Miranda I.M. (2015). Fluconazole and voriconazole resistance in Candida parapsilosis is conferred by gain-of-function mutations in MRR1 transcription factor gene. Antimicrob. Agents Chemother..

[188] Branco J., Ola M., Silva R.M., Fonseca E., Gomes N.C., Martins-Cruz C., Silva A.P., Silva-Dias A., Pina-Vaz C., Erraught C. (2017). Impact of ERG3 mutations and expression of ergosterol genes controlled by UPC2 and NDT80 in Candida parapsilosis azole resistance. Clin. Microbiol. Infect..

[189] Garcia-Effron G., Katiyar S.K., Park S., Edlind T.D., Perlin D.S. (2008). A naturally occurring proline-to-alanine amino acid change in Fks1p in Candida parapsilosis, Candida orthopsilosis, and Candida metapsilosis accounts for reduced echinocandin susceptibility. Antimicrob. Agents Chemother..

[190] Chiotos K., Vendetti N., Zaoutis T.E., Baddley J., Ostrosky-Zeichner L., Pappas P., Fisher B.T. (2016). Comparative effectiveness of echinocandins versus fluconazole therapy for the treatment of adult candidaemia due to Candida parapsilosis: A retrospective observational cohort study of the Mycoses Study Group (MSG-12). J. Antimicrob. Chemother..

[191] Fernández-Ruiz M., Aguado J.M., Almirante B., Lora-Pablos D., Padilla B., Puig-Asensio M., Montejo M., García-Rodríguez J., Pemán J., Ruiz Pérez de Pipaón M. (2014). Initial use of echinocandins does not negatively influence outcome in Candida parapsilosis bloodstream infection: A propensity score analysis. Clin. Infect. Dis..

[192] Satoh K., Makimura K., Hasumi Y., Nishiyama Y., Uchida K., Yamaguchi H. (2009). Candida auris sp. nov., a novel ascomycetous yeast isolated from the external ear canal of an inpatient in a Japanese hospital. Microbiol. Immunol..

[193] Chow N.A., De Groot T., Badali H., Abastabar M., Chiller T.M., Meis J.F. (2019). Potential fifth clade of Candida auris, Iran, 2018. Emerg. Infect. Dis..

[194] van Schalkwyk E., Mpembe R.S., Thomas J., Shuping L., Ismail H., Lowman W., Karstaedt A.S., Chibabhai V., Wadula J., Avenant T. (2019). Epidemiologic shift in candidemia driven by Candida auris, South Africa, 2016–2017. Emerg. Infect. Dis..

[195] Mathur P., Hasan F., Singh P.K., Malhotra R., Walia K., Chowdhary A. (2018). Five-year profile of candidaemia at an Indian trauma centre: High rates of Candida auris blood stream infections. Mycoses.

[196] Escandón P., Chow N.A., Caceres D.H., Gade L., Berkow E.L., Armstrong P., Rivera S., Misas E., Duarte C., Moulton-Meissner H. (2019). Molecular epidemiology of Candida auris in Colombia reveals a highly related, countrywide colonization with regional patterns in amphotericin B resistance. Clin. Infect. Dis..

[197] Welsh R.M., Bentz M.L., Shams A., Houston H., Lyons A., Rose L.J., Litvintseva A.P. (2017). Survival, persistence, and isolation of the emerging multidrug-resistant pathogenic yeast Candida auris on a plastic health care surface. J. Clin. Microbiol..

[198] Ledwoch K., Maillard J.Y. (2018). Candida auris dry surface biofilm (DSB) for disinfectant efficacy testing. Materials.

[199] Casadevall A., Kontoyiannis D.P., Robert V. (2019). On the emergence of Candida auris: Climate change, azoles, swamps, and birds. mBio.

[200] Jackson B.R., Chow N., Forsberg K., Litvintseva A.P., Lockhart S.R., Welsh R., Vallabhaneni S., Chiller T. (2019). On the origins of a species: What might explain the rise of Candida auris?. J. Fungi.

[201] Chow N.A., Gade L., Tsay S.V., Forsberg K., Greenko J.A., Southwick K.L., Barrett P.M., Kerins J.L., Lockhart S.R., Chiller T.M. (2018). Multiple introductions and subsequent transmission of multidrug-resistant Candida auris in the USA: A molecular epidemiological survey. Lancet Infect. Dis..

[202] Rybak J.M., Doorley L.A., Nishimoto A.T., Barker K.S., Palmer G.E., Rogers P.D. (2019). Abrogation of triazole resistance upon deletion of CDR1 in a clinical isolate of Candida auris. Antimicrob. Agents Chemother..

[203] Ben-Ami R., Berman J., Novikov A., Bash E., Shachor-Meyouhas Y., Zakin S., Maor Y., Tarabia J., Schechner V., Adler A. (2017). Multidrug-resistant Candida haemulonii and C. auris, Tel Aviv, Israel. Emerg. Infect. Dis..

[204] Kim S.H., Iyer K.R., Pardeshi L., Muñoz J.F., Robbins N., Cuomo C.A., Wong K.H., Cowen L.E. (2019). Genetic analysis of Candida auris implicates Hsp90 in morphogenesis and azole tolerance and Cdr1 in azole resistance. mBio.

[205] Kean R., Delaney C., Sherry L., Borman A., Johnson E.M., Richardson M.D., Rautemaa-Richardson R., Williams C., Ramage G. (2018). Transcriptome assembly and profiling of Candida auris reveals novel insights into biofilm-mediated resistance. mSphere.

[206] Rybak J.M., Muñoz J.F., Barker K.S., Josie E., Parker J.E., Esquivel B.D., Elizabeth L., Berkow E.L., Lockhart S.L., Gade L. (2020). Mutations in TAC1B: A novel genetic determinant of clinical fluconazole resistance in Candida auris. mBio.

[207] Sekizuka T., Iguchi S., Umeyama T., Inamine Y., Makimura K., Kuroda M., Miyazaki Y., Kikuchi K. (2019). Clade II Candida auris possess genomic structural variations related to an ancestral strain. PLoS ONE.

[208] Alastruey-Izquierdo A., Alcazar-Fuoli L., Rivero-Menéndez O., Ayats J., Castro C., García-Rodríguez J., Goterris-Bonet L., Ibáñez-Martínez E., Linares-Sicilia M.J., Martin-Gomez M.T. (2018). Molecular identification and susceptibility testing of molds isolated in a prospective surveillance of triazole resistance in Spain (FILPOP2 Study). Antimicrob. Agents Chemother..

[209] Fischer J., van Koningsbruggen-Rietschel S., Rietschel E., Vehreschild M.J.G.T., Wisplinghoff H., Krönke M., Hamprecht A. (2014). Prevalence and molecular characterization of azole resistance in Aspergillus spp. isolates from German cystic fibrosis patients. J. Antimicrob. Chemother..

[210] Lestrade P.P.A., Meis J.F., Melchers W.J.G., Verweij P.E. (2019). Triazole resistance in Aspergillus fumigatus: Recent insights and challenges for patient management. Clin. Microbiol. Infect..

[211] Meneau I., Sanglard D. (2005). Azole and fungicide resistance in clinical and environmental Aspergillus fumigatus isolates. Med. Mycol..

[212] van der Linden J.W.M., Arendrup M.C., Warris A., Lagrou K., Pelloux H., Hauser P.M., Chryssanthou E., Mellado E., Kidd S.E., Tortorano A.M. (2015). Prospective multicenter international surveillance of azole resistance in Aspergillus fumigatus. Emerg. Infect. Dis..

[213] Perlin D.S., Rautemaa-Richardson R., Alastruey-Izquierdo A. (2017). The global problem of antifungal resistance: Prevalence, mechanisms, and management. Lancet Infect. Dis..

[214] Fraczek M.G., Bromley M., Buied A., Moore C.B., Rajendran R., Rautemaa R., Ramage G., Denning D.W., Bowyer P. (2013). The cdr1B efflux transporter is associated with non-cyp51a-mediated itraconazole resistance in Aspergillus fumigatus. J. Antimicrob Chemother..

[215] Parent-Michaud M., Dufresne P.J., Fournier E., Folch B., Martineau C., Moreira S., Doucet N., Repentigny L.D., Dufresne S.F. (2020). Prevalence and mechanisms of azole resistance in clinical isolates of Aspergillus section Fumigati species in a Canadian tertiary care centre, 2000 to 2013. J. Antimicrob Chemother..

[216] Rybak J.M., Ge W., Wiederhold N.P., Parker J.E., Kelly S.L., Rogers D.P., Fortwendel J.R. (2019). Mutations in hmg1, Challenging the Paradigm of Clinical Triazole Resistance in Aspergillus fumigatus. mBio.

[217] Camps S.M.T., Dutilh B.E., Arendrup M.C., Rijs A.J.M.M., Snelders E., Huynen M.A., Verweij P.E., Melchers W.J.G. (2012). Discovery of a hapE mutation that causes azole resistance in Aspergillus fumigatus through whole genome sequencing and sexual crossing. PLoS ONE.

[218] Alcazar-Fuoli L., Mellado E., Alastruey-Izquierdo A., Cuenca-Estrella M., Rodriguez-Tudela J.L. (2008). Aspergillus section Fumigati: Antifungal susceptibility patterns and sequence-based identification. Antimicrob. Agents Chemother..

[219] Lamoth F. (2016). Aspergillus fumigatus-related species in clinical practice. Front. Microbiol..

[220] Satish S., Jiménez-Ortigosa C., Zhao Y., Hee Lee M., Dolgov E., Krüger T., Park S., Denning D.W., Kniemeyer O., Brakhage A.A. (2019). Stress-Induced Changes in the Lipid Microenvironment of β-(1,3)-d-Glucan Synthase Cause Clinically Important Echinocandin Resistance in Aspergillus fumigatus. mBio.

[221] Risslegger B., Zoran T., Lackner M., Aigner M., Sánchez-Reus F., Rezusta A., Chowdhary A., Taj-Aldeen S.J., Arendrup M.C., Oliveri S. (2017). A prospective international Aspergillus terreus survey: An EFISG, ISHAM and ECMM joint study. Clin. Microbiol. Infect..

[222] Vahedi Shahandashti R., Lass-Flörl C. (2019). Antifungal resistance in Aspergillus terreus: A current scenario. Fungal Genet. Biol..

[223] Posch W., Blatzer M., Wilflingseder D., Lass-Flörl C. (2018). Aspergillus terreus: Novel lessons learned on amphotericin B resistance. Med. Mycol..

[224] Zoran T., Sartori B., Sappl L., Aigner M., Sánchez-Reus F., Rezusta A., Chowdhary A., Taj-Aldeen S.J., Arendrup M.C., Oliveri S. (2018). Azole-resistance in Aspergillus terreus and related species: An emerging problem or a rare phenomenon?. Front. Microbiol..

[225] Mellado E., Garcia-Effron G., Alcazar-Fuoli L., Cuenca-Estrella M., Rodriguez-Tudela J.L. (2004). Substitutions at methionine 220 in the 14alpha-sterol demethylase (cyp51A) of Aspergillus fumigatus are responsible for resistance in vitro to azole antifungal drugs. Antimicrob. Agents Chemother..

[226] Pelaez T., Gijón P., Bunsow E., Bouza E., Sánchez-Yebra W., Valerio M., Gama B., Cuenca-Estrella M., Mellado E. (2012). Resistance to voriconazole due to a G448S substitution in Aspergillus fumigatus in a patient with cerebral aspergillosis. J. Clin. Microbiol..

[227] Snelders E., Karawajczyk A., Schaftenaar G., Verweij P.E., Melchers W.J.G. (2010). Azole resistance profile of amino acid changes in Aspergillus fumigatus CYP51A based on protein homology modeling. Antimicrob. Agents Chemother..

[228] Tashiro M., Izumikawa K., Hirano K., Ide S., Mihara T., Hosogaya N., Takazono T., Morinaga Y., Nakamura S., Kurihara S. (2012). Correlation between triazole treatment history and susceptibility in clinically isolated Aspergillus fumigatus. Antimicrob. Agents Chemother..

[229] van Ingen J., van der Lee H.A., Rijs T.A.J., Zoll J., Leenstra T., Melchers W.J.G., Verweij P.E. (2015). Azole, polyene and echinocandin MIC distributions for wild-type, TR34/L98H and TR46/Y121F/T289A Aspergillus fumigatus isolates in the Netherlands. J. Antimicrob. Chemother..

[230] Bader O., Weig M., Reichard U., Lugert R., Kuhns M., Christner M., Held J., Peter S., Schumacher U., Buchheidt D. (2013). Cyp51A-based mechanisms of Aspergillus fumigatus azole drug resistance present in clinical samples from Germany. Antimicrob. Agents Chemother..

[231] Bromley M., Johns A., Davies E., Fraczek M., Gilsenan J.M., Kurbatova N., Keays M., Kapushesky M., Gut M., Gut I. (2016). Mitochondrial complex I is a global regulator of secondary metabolism, virulence and azole sensitivity in fungi. PLoS ONE.

[232] Deng S., Zhang L., Ji Y., Verweij P.E., Tsui K.M., Hagen F., Houbraken J., Meis J.F., Abliz P., Wang X. (2017). Triazole phenotypes and genotypic characterization of clinical Aspergillus fumigatus isolates in China. Emerg. Microbes Infect..

[233] Denning D.W., Park S., Lass-Flörl C., Fraczek M.G., Kirwan M., Gore R., Smith J., Bueid A., Moore C.B., Bowyer P. (2011). High-frequency triazole resistance found in nonculturable Aspergillus fumigatus from lungs of patients with chronic fungal disease. Clin. Infect. Dis..

[234] Diaz-Guerra T.M., Mellado E., Cuenca-Estrella M., Rodriguez-Tudela J.L. (2003). A point mutation in the 14alpha-sterol demethylase gene cyp51a contributes to itraconazole resistance in Aspergillus fumigatus. Antimicrob. Agents Chemother..

[235] Hodiamont C.J., Dolman K.M., Ten Berge I.J.M., Melchers W.J.G., Verweij P.E., Pajkrt D. (2009). Multiple-azole-resistant Aspergillus fumigatus osteomyelitis in a patient with chronic granulomatous disease successfully treated with long-term oral posaconazole and surgery. Med. Mycol..

[236] Howard S.J., Cerar D., Anderson M.J., Albarrag A., Fisher M.C., Pasqualotto A.C., Laverdiere M., Arendrup M.C., Perlin D.S., Denning D.W. (2009). Frequency and evolution of azole resistance in Aspergillus fumigatus associated with treatment failure. Emerg. Infect. Dis..

[237] Brauer V.S., Rezende C.P., Pessoni A.M., De Paula R.G., Rangappa K.S., Nayaka S.C., Gupta V.K., Almeida F. (2019). Antifungal agents in agriculture: Friends and foes of public health. Biomolecules.

[238] Zhang J., Snelders E., Zwaan B.J., Schoustra S.E., Meis J.F., van Dijk K., Hagen F., van der Beek M.T., Kampinga G.A., Zoll J. (2019). A novel environmental azole resistance mutation in Aspergillus fumigatus and a possible role of sexual reproduction in its emergence. mBio.

[239] Verweij P.E., Mellado E., Melchers W.J. (2007). Multiple-triazole–resistant aspergillosis. N. Engl. J. Med..

[240] Hare R.K., Gertsen J.B., Astvad K.M.T., Degn K.B., Løkke A., Stegger M., Andersen P.S., Kristensen L., Arendrup M.C. (2019). In vivo selection of a unique tandem repeat mediated azole resistance mechanism (TR120) in Aspergillus fumigatus cyp51A, Denmark. Emerg. Infect. Dis..

[241] Perlin D.S., Wiederhold N.P. (2017). Culture-independent molecular methods for detection of antifungal resistance mechanisms and fungal identification. J. Infect. Dis..

[242] Gabaldón T., Consortium OPATHY (2019). Recent trends in molecular diagnostics of yeast infections: From PCR to NGS. FEMS Microbiol. Rev..

[243] Arastehfar A., Wickes B.L., Ilkit M., Pincus D.H., Daneshnia F., Pan W., Fang W., Boekhout T. (2019). Identification of mycoses in developing countries. J. Fungi.

